# Effective Non-Viral Delivery of siRNA to Acute Myeloid Leukemia Cells with Lipid-Substituted Polyethylenimines

**DOI:** 10.1371/journal.pone.0044197

**Published:** 2012-08-31

**Authors:** Breanne Landry, Hamidreza Montazeri Aliabadi, Anuja Samuel, Hilal Gül-Uludağ, Xiaoyan Jiang, Olaf Kutsch, Hasan Uludağ

**Affiliations:** 1 Department of Chemical & Materials Engineering, Faculty of Engineering, University of Alberta, Edmonton, Alberta, Canada; 2 Department of Biomedical Engineering, Faculty of Medicine & Dentistry, University of Alberta, Edmonton, Alberta, Canada; 3 Terry Fox Laboratories, BC Cancer Agency, Vancouver, British Columbia, Canada; 4 Centre for AIDS Research, University of Alabama at Birmingham, Birmingham, Alabama, United States of America; 5 Faculty of Pharmacy and Pharmaceutical Sciences, University of Alberta, Edmonton, Alberta, Canada; National Institutes of Health, United States of America

## Abstract

Use of small interfering RNA (siRNA) is a promising approach for AML treatment as the siRNA molecule can be designed to specifically target proteins that contribute to aberrant cell proliferation in this disease. However, a clinical-relevant means of delivering siRNA molecules must be developed, as the cellular delivery of siRNA is problematic. Here, we report amphiphilic carriers combining a cationic polymer (2 kDa polyethyleneimine, PEI2) with lipophilic moieties to facilitate intracellular delivery of siRNA to AML cell lines. Complete binding of siRNA by the designed carriers was achieved at a polymer:siRNA ratio of ∼0.5 and led to siRNA/polymer complexes of ∼100 nm size. While the native PEI2 did not display cytotoxicity on AML cell lines THP-1, KG-1 and HL-60, lipid-modification on PEI2 slightly increased the cytotoxicity, which was consistent with increased interaction of polymers with cell membranes. Cellular delivery of siRNA was dependent on the nature of lipid substituent and the extent of lipid substitution, and varied among the three AML cell lines used. Linoleic acid-substituted polymers performed best among the prepared polymers and gave a siRNA delivery equivalent to better performing commercial reagents. Using THP-1 cells and a reporter (GFP) and an endogenous (CXCR4) target, effective silencing of the chosen targets was achieved with 25 to 50 nM of siRNA concentrations, and without adversely affecting subsequent cell growth. We conclude that lipid-substituted PEI2 can serve as an effective delivery of siRNA to leukemic cells and could be employed in molecular therapy of leukemia.

## Introduction

Acute myeloid leukemia (AML) is the most common acute leukemia in adults, with an estimated >13,000 new cases yearly and a mortality rate of ∼10,000 in the US alone [1]. Development of novel AML therapies is urgently needed due to poor prognosis of the disease with a five-year survival rate of 30% for younger adults and ∼15% for elderly patients [2]. Only in childhood AML, ∼60% of patients can be cured of AML with very intensive chemotherapy [3]. The chemotherapy remains the front-line treatment, but alternative therapeutic approaches are required due to high relapse rates and limited treatment options for patients that cannot bear the toxic side-effects of chemotherapy [4]. Chemotherapy also leads to long-term undesired consequences; ∼66% of survivors have either a chronic or late-effect due to cancer treatment and ∼33% of these effects are considered major, serious or life threatening [1]. With better understanding of molecular changes in malignant transformations, treatments that target tumor-specific changes will lead to more effective therapies as the normal cells transform into malignant cells. Towards this end, a highly specific leukemia therapy can be developed by exploiting the RNA interference (RNAi) to silence the aberrant protein(s) responsible for the disease [5], [6].

There are two main approaches for RNAi, using either a plasmid encoding for short hairpin RNA (shRNA) or delivering small interfering RNA (siRNA) where the shRNA transcription and processing steps can be omitted [7]. The use of siRNA is a more practical approach bypassing the need to express the shRNA at sufficient quantities in hard-to-transfect primary cells. In cytosol, the siRNA duplexes assemble into a pre-RISC (RNA-induced silencing complex) containing specific proteins, including argonaute proteins (AGO1, 3 or 4) [8], [9]. The siRNA duplexes become unwound in AGOs, where the guiding strand directs the mature-RISC to target desired mRNA based on complementary base pairing [8]. Endonucleoyltic cleavage and/or translational repression of the mRNA [8], [9] subsequently silences the desired protein target. Delivery systems, however, are an absolute necessity for effective use of siRNA since the molecules are highly sensitive to serum nucleases and their large (∼13 kDa) and anionic nature (due to its phosphodiesterase backbone) prevents siRNA to traverse cellular membranes. Cationic biomolecules capable of binding and neutralizing the anionic charges of siRNA and packaging the siRNA into nano-sized complexes can serve as effective siRNA carriers [10]. The utility of cationic carries for siRNA therapy in AML has been explored as early as 2003, where Raf-1 and Bcl-2 proteins were suppressed in AML cells by using the synthetic carrier Oligofectamine™. However, the resulting apoptotic response required 400 nM siRNA [5], a concentration too high for practical applications. It was evident that a more efficient delivery system was required to advance siRNA therapy for AML. Recent RNAi delivery attempts in leukemia cells have employed a variety of commercial carriers, which included; (i) Lipofectamine™ 2000 in chronic myeloid leukemia (CML) K562 cells, and AML cells (KG-1/HL-60/U937/primary) [11]–[16], (ii) RNAiMAX™ in K562 [17], (iii) HiPerFect™ in K562 and T-ALL (Jurkat) cells [18], [19], (iv) DOTAP in *Bcr-abl* positive CML cells (2Dp210-modified/patient samples) [20], (v) Lipofectin in myeloid neoplasm cells (HMC-1) [21] (vi) and Oligofectamine™ in T-cell lympoblastic leukemia cells (CCRF-CEM) [21]. Other carriers used for siRNA delivery were cell penetrating peptides (Tat–LK15 peptide in K562 cells) [16], CADY peptide in THP-1 cells [23], and functionalized carbon nanotubes in K562 cells [24]. Many of the above studies focused on discovery of possible targets for silencing and/or mechanisms of drug action, without pursuing siRNA delivery as a therapy. A systematic analysis of carrier features responsible for effective siRNA delivery was not conducted, which is critical for design of more effective siRNA carriers suitable for clinical use.

We previously reported on siRNA delivery by amphiphilic cationic polymers with lipid substituents to anchorage-dependent malignant cells [25]. The polymers provided the necessary cationic charge for siRNA binding whereas the lipid component provided the hydrophobicity for improved interactions with cellular membranes. The polymeric component was derived from polyethylenimine (PEI), whose prototypical member, 25 kDa branched PEI (PEI25), is widely used as an effective transfection agent [26]–[28]. Since the cytotoxicity of PEI25 has been a major impediment for its therapeutic use, we employed a smaller PEI (2 kDa; PEI2) as the polymer backbone since it displays minimal cytotoxicity [29]–[34]. Although PEI2 displays effective binding to nucleic acids in buffers, the resultant complexes were ineffective for nucleic acid delivery into cells. Lipid substitution on PEI2 enhanced the assembly of nucleic acids into nano-particles, improved the cellular uptake and, depending on structural features of lipid substituents, enabled silencing of selected molecular targets in breast cancer cells [25], [35]. Leukemic cells, on the other hand, are structurally different from anchorage-dependent cells, with minimal surface area and endocytic activity, and are known to be difficult to transfect (as discussed in [36]).

This study explored the utility of lipid-substituted polymers for siRNA delivery to leukemic cells. It was our aim to determine the relative effectiveness of these carriers for siRNA delivery and to elucidate carrier features critical for delivery. We focused on AML subgroup of leukemia and employed 3 well-characterized cell models (THP-1, KG-1 and HL-60 cells). The PEI25 was employed as a reference reagent, given its prominent use for siRNA delivery to anchorage-dependent cells. A systematic approach was employed to investigating the role of lipid substitution as well as the nature of substituted lipid on siRNA binding, toxicity, and siRNA delivery and silencing. The results showed that (i) PEI25 was not effective in siRNA delivery to leukemic cells unlike the anchorage-dependent cells, and (ii) lipid substitution improved the siRNA delivery of cationic polymers, and (iii) effective silencing could be obtained at clinically acceptable siRNA doses (20–50 nM). These results provide encouraging data to pursue the described carriers for siRNA-based molecular therapy of leukemia.

## Materials and Methods

### 2.1 Materials

PEI25 (M_n_: 10 kDa, M_w_: 25 kDa) and PEI2 (M_n_: 1.8 kDa, M_w_: 2 kDa), anhydrous dimethyl sulfoxide (DMSO), myristoyl chloride (C14; 97%), palmitoyl chloride (C16; 98%), octanoyl chloride (C18∶1 9Z; 99%), linoleoyl acid (C18∶2 9Z, 12Z; 99%), 3-(4,5-demethyl-2-thiazoylyl)-2,5-diphenyl-2H-tetrazolium bromide (MTT), *N*-[1-(2,3-dioleoyloxy)]-N,N,N-trimethylammonium propane methylsulfate (DOTAP), trypan blue solution (0.4%), and heparin sodium from porcine intestinal mucosa were purchased from Sigma-Aldrich (St. Louis, MO). Stearoyl chloride (C18; >98.5%) was obtained from FLUKA. Clear filtered HBSS (phenol red free) was prepared in-house. Unlabeled negative control siRNA, 5′-carboxyfluorescein (FAM)-labelled negative control siRNA, GFP siRNA (GFP-22) and CXCR4 siRNA (HSC.RNAI.N001008540.12.1) were purchased from Ambion (Austin, TX), Shanghai GenePharma Co., Ltd (Shanghai, China) and Qiagen (Toronto, ON) and IDT (Coralville, IA), respectively. Hanks Balanced Salt Solution (HBSS), Dulbecco’s modified Eagle medium (DMEM; low glucose with L-glutamine; 11885), and RPMI Medium 1640 with L-glutamine (11835), opti-MEM® I reduced serum medium (31985), penicillin (10000 U/mL), and streptomycin (10 mg/mL) were from Invitrogen (Grand Island, NY). Fetal bovine serum (FBS; A15-751) was purchased from PAA Laboratories Inc. (Etobicoke, ON). RNAi-mate was obtained from Shanghai GenePharma Co., Ltd, Lipofectamine™ 2000 and Lipofectamine™ RNAiMAX Reagent from Invitrogen, Metafectamine Pro from Biontex (San Diego, California) and FuGENE® HD from Roche (Laval, QC) and HiPerFect Transfection Reagent from Qiagen (Mississauga, Ontario).

### 2.2 Cell Models and Culture

The cell lines THP-1, KG-1 and HL-60 cells used as the AML models were obtained from the American Type Culture Collection (Manassas, VA). THP-1 and KG-1 cell were maintained in RPMI medium and HL-60 cells were maintained in DMEM Low Glucose medium, all containing 10% FBS (heat inactivated at 56°C for 30 min) and 1% penicillin/streptomycin under normal conditions (37°C, 5% CO_2_ under humidified atmosphere). The cells were maintained at concentrations between 0.1×10^5^ and 4×10^5^ cells/ml (monitored by hematocytometer cell counts) and by weekly passage by dilution after removing the spent medium with centrifugation at 600 rpm (72 g) for 5 min. To obtain Green Fluorescent Protein expressing THP-1 cells, a retroviral vector expressing enhanced GFP (EGFP) was generated by cloning EGFP into pMSCVpuro (Invitrogen). The murine stem cell virus-based vector was chosen as it provides relatively stable long-term expression of the transgene and is less prone to transcriptional shutdown in THP-1 cells than other retroviral vector systems tested. To generate retroviral particles, pMSCV-EGFP was transfected into 293T cells with Fugene HD. Gag/pol were provided in trans and VSV-G was utilized as viral coat protein. Retroviral supernatants were harvested 24 h post transfection and used to transduce THP-1 cells. The cells were then selected using puromycin and further enriched for EGFP expression using fluorescence activated cell sorting. The resulting GFP-expressing THP-1 cells were cultured as above.

### 2.3 Synthesis of Lipid-Substituted Polymers

The PEI2 polymers substituted with lipids (caprylic acid; CA, palmitic acid; PA, oleic acid; OA, linoleic acid; LA; stearic acid; StA, myristic acid; MA) were prepared in house, where the synthesis and characterization have been previously described [37], [38]. Briefly, a 2 kDa PEI solution (50% in water) was first purified by freeze-drying. Commercially available lipid chlorides (CA, PA, OA, LA, StA and MA) were then substituted by N-acylation of PEI onto the amine groups by addition of the lipid chlorides to 100 mg of PEI in DMS0 for 24 h at ambient temperature under argon. To produce a range of substitution levels for each lipid, four different feed ratios were utilized (lipid:polymer  = 0.012, 0.066, 0.1 and 0.2) and the polymers were precipitated and washed with excess ethyl ether. The lipid-substituted polymers were dried under vacuum at ambient temperature over night. The substitution was analysed by ^1^H-NMR (Bruker 300 MHz; Billerica, MA) in D_2_O). The characteristic proton shifts of lipids (δ∼0.8 ppm; -CH_3_) and PEI (δ∼2.5–2.8 ppm; NH-CH_2_-CH_2_-NH-) were integrated and normalised to the number of protons in each peak in order to calculate the lipid substitution levels. **Table S1** summarizes the employed feed ratios as well as the final level of lipid substitutions obtained. The numbers of lipid methylenes substituted in each polymer were calculated by multiplying the level of lipid substitution (from ^1^H-NMR) with the number of methylenes in each lipid. Percent lipid substitution was calculated by dividing the number of lipid substituted with the number of amines available in each PEI2 (14).

### 2.4 siRNA/Polymer Complex Formation

Polymer/siRNA complexes were formed by first adding a desired amount of siRNA (0.37 µg; 25 nM final siRNA well concentration) to 150 mM NaCl solution. The polymers (PEI25, PEI2 and lipid-substituted PEI2s; all dissolved in ddH_2_O) were then added to the siRNA solutions at desired polymer:siRNA ratios (8∶1, 4∶1 and 2∶1 w/w, corresponding to 63.2∶1, 31.6∶1, 15.8∶1 N/P ratios), bringing the volume to 30 µL. After mixing, the complexes were incubated at room temperature for 30 min before addition (triplicate; 10 µL/well) to the cells (note that 30 minutes of complex incubation was within the optimal range for siRNA delivery; **Figure S1)**. For electron microscopy imaging, complexes were prepared in the same manner, except the 150 mM NaCl solution was replaced with ultra pure water to prevent NaCl crystal formation. After 30 min of incubation, 5 µL of complex solution was transferred to a 3 mm Formvar film coated grid. The grid was allowed to dry (∼20 min) and complexes were imaged by a Philips/FEI (Morgagni) Transmission Electron Microscope with CCD camera (TEM-CCD).

### 2.5 Cytotoxicity

The extent of the polymer/siRNA complex cytotoxicity was determined by the MTT assay. The complexes were prepared at 8∶1 w/w ratio as described above. The cells were seeded in 24-well plates with 0.5 mL medium per well and allowed to acclimatize for 24 h. The complexes were then added in triplicate for final concentrations of 1.25, 2.5, 5 and 10 µg/mL and incubated for 24 h under normal maintenance conditions. The MTT solution (40 µL, 4 mg/mL in HBSS) was then added to each well and the cells were incubated for 2 h. The plates were centrifuged, the medium removed, and 200 µL of DMSO was added to each well to dissolve the MTT crystals formed. The optical density of the solutions (570 nm) was measured by an ELx800 Universal Microplate Reader (BioTek Instruments; Winooski, VT, USA). Background was determined with medium only wells and subtracted from the obtained optical densities. The percentage of cell viability was calculated as follows: 100% x (absorbance of polymer treated cells/absorbance of untreated cells).

### 2.6 Analysis of siRNA Binding to Polymers

Gel electrophoresis was performed for assessment of siRNA binding efficiency of polymers, as well as for dissociation of siRNA/polymer complexes with heparin. For the binding studies, 3 µL of 0.1 mg/mL control siRNA (in ddH_2_O) was incubated with various concentrations of polymers (in ddH_2_0) in 25 µL of 150 mM NaCl for 20 min to form complexes. Loading dye (4 µL, 6x, 40% sucrose with bromophenol blue/xylene cyanole) was added to samples and the samples were run on a 0.8% agarose gel containing 1 µg/mL ethidium bromide (130 V for 20 min). The gels were visualized under UV illumination and bands corresponding to free siRNA were quantified by spot densitometer. siRNA alone was run as a reference control (i.e., 0% binding). Percent binding (%Binding) was calculated as: 100% × [(control siRNA − free siRNA) ÷ control siRNA]. Percent binding was plotted as a function of polymer concentration and concentration required for 50% binding of siRNA (BC_50_) was estimated based on sigmoidal curve fits.

### 2.7 siRNA Delivery to Leukemia Cells

Effectiveness of carriers for siRNA delivery was determined by measuring the percentage of cells positive for siRNA and mean fluorescence of cells after delivery of FAM-labelled control (scrambled) siRNA (CsiRNA-F). To account for cellular auto-fluorescence due to complex exposure, a non-labelled scrambled (control) siRNA (CsiRNA) was utilized as a control for each siRNA-polymer complex prepared. In cases where the results from CsiRNA are not shown, the autofluorescence was found to be insignificant. THP-1, KG-1 or HL-60 cells were seeded in 24-well plates (0.35 mL fresh medium/well) and allowed to acclimatize for 24 h in normal maintenance conditions. The siRNA-polymer complexes were prepared as in Section 2.4 and 10 µl of complex solution was slowly added to each well containing the cells (0.35 mL medium/well in triplicate). A 30 min of complex formation between the siRNA and the polymers was found to give the optimal uptake (**Figure S1**), so that complexes were exposed to the cells after this incubation time. At indicated time points (see figure legends), the cells were transferred to eppendorf tubes and centrifuged (1200 rpm; 100 rcf). Cells were washed with clear HBSS, resuspended in 100 µl of clear HBSS and then fixed with 3.7% formalin in HBSS. The siRNA delivery to the cells was assessed by flow cytometry (Cell Lab Quanta™ SC; Beckman Couter) using the FL-1 detection channel, fluorescence plate reader at λ_EX_ of 485 nm and λ_EM_ of 527 nm (Fluroskan Ascent, Thermo Labsystems), or by epifluorescence microscopy (FSX100; Olympus) as elaborated in the figures.

Competitive inhibition studies were performed by incubating the cells with free lipids (LA, OA, StA; 0–100 µM) followed by treatment of the cells with FAM-labelled siRNA/polymer complexes. Effect of serum on complex delivery was also determined; the percentage of FBS in medium was varied between 0 and 50% prior to complex treatment and cell uptake was determined by flow cytometry as described above.

For internalization studies, siRNA delivery was performed as described above with the following modifications. Delivery was performed at both 4 and 37°C from 1 to 24 h and subsequently split into trypan blue treated and non-treated groups. For the 4°C groups, cells were placed at 4°C, 20 min prior to addition of complexes and immediately put on ice in subsequent steps. At each time point, cells were transferred to 1.5 ml tubes, centrifuged and the medium was removed. Each group was split into a trypan blue and a without trypan blue group. 100 µl of 0.4% trypan blue in HBSS (or HBSS) was added to each tube (containing 100 µl medium) and cells were resuspended and incubated for 5 min. They were then fixed with 3.7% formalin and washed twice with 1 ml HBSS (to remove trypan blue) prior to flow cytometry.

A comparison between lipid-substituted polymers and commercial reagents (RNA-mate, Lipofectamine™ 2000, RNAiMAX™, Metafectamine, DOTAP, Fugene HD and HiPerFect) was performed by delivering siRNA complexes (24 h) prepared with CsiRNA-F. Complexes were prepared as closely as possible to the manufactures directions while maintaining a consistency necessary for comparison. The incubation time with the cells, medium volume and type and serum concentrations were all standardized. As most vendors suggest the use of OPTI-MEM for complex preparation, the complexes were prepared with OPTI-MEM as the buffer. siRNA (0.37 µg) and desired reagents was added to 150 µL OPTI-MEM solutions separately. The reagent amounts were 2.9 µL (1 mg/ml) for PEI25 and PEI2-LA20, 5.9 µL for RNAi-mate, 4 µL for Lipofectamine™ 2000, 7.5 µL for RNAiMAX™ (pre-diluted 1∶4 in OPTI-MEM), 1.8 µL for Metafactamine, 4 µL for Fugene HD, 2.2 µl for DOTAP (1 mg/mL), and 4.5 µL for HiPerFect (pre-diluted 1∶4 in OPTI-MEM). The amount of the reagents was halved for low concentration experiment. The siRNA and reagent solutions were then vortexed, except Metafectamine that was mixed by pipetting once. The siRNA-reagent solutions was then mixed by gently vortexing except for DOTAP which was mixed by pipetting and Metafectamine which was not mixed. PEI25, PEI2-LA20 and RNAi-mate complexes were incubated for 30 min, Lipofectamine™ 2000, RNAiMAX™ and Metafectamine were incubated for 20 minutes, Fugene HD and DOTAP were incubated for 15 min and HiPerFect was incubated for 10 min, prior to drop-wise addition (100 µL) to cells in 200 µL of RPMI medium. The commercial reagents were ranked (from 1 to 9; 1 being the best and 9 being the worst) according to siRNA uptake results from flow cytometry, based on percentage of cell population positive for siRNA and mean siRNA fluorescence/cell. If reagents had comparable fluorescence levels (due to overlapping SDs), their ranks were averaged and each was given the same mean value. The ranking was then averaged over the three cell lines to provide an overall performance ranking.

### 2.8 GFP Silencing in THP-1 Cells

GFP-expressing THP-1 cells were used as a model for silencing studies. siRNA complex formation and delivery to the cells was performed as described in Section 2.4 and 2.7, utilizing GFP specific siRNA (GFP-siRNA) and scrambled siRNA (CsiRNA). For the time course studies, cells were treated with desired siRNAs continuously during the experimental duration; cells were subcultured every 3 days to prevent over-growth. Subculturing was performed by dilution (x10) into fresh medium after resuspension. All groups were subcultured with the same ratio regardless of cell concentration to ensure that the concentration of any remaining complexes stayed constant. For studies including the commercial reagents, selected reagent preparation was performed with OPTI-MEM as described in Section 2.7 for the commercial reagent delivery comparison study, keeping the same reagent:siRNA ratios (high ratios) and at siRNA concentration of 50 nM. GFP silencing was assessed by flow cytometry after cell fixation (as described in Section 2.7) using the FL-1 detection channel. Percent decrease in mean fluorescence was calculated as follows: 100 - {[Mean FL1 of cells treated with GFP-siRNA/polymer complexes ]/[Mean FL1 of cells treated with CsiRNA/polymer complexes] x 100%}. Percent decrease in GFP-positive cells was calculated as follows: [% of GFP-negative cells of GFPsiRNA/polymer treated cells] - [% of GFP-negative cells of CsiRNA/polymer treated cells]. Gating was performed as described in **Section 3.7**.

For studies where GFP silencing was followed at the mRNA level, total RNA was extracted from treated THP-1 cells in 12-well plates (biological duplicates) with the RNAeasy Mini Kit (Qiagen). The extracted RNA was then quantified by spectrophotometry (GE Nanovue). cDNA was synthesised following Invitrogen’s protocol, briefly adding 2 µL master mix 1 (0.5 µL Oligo(dT)_12–18_ Primer, 0.5 µL random primers and 1 µL (10 mM) MdNTP’s per sample) to 10 µL of RNA (2500 ng) and then heated at 65°C for 5 min. 7 µL of Master Mix 2 (4 µL 5 × Synthesis Buffer, 2 µL DTT (0.1 M) and 1 µL RNAout RNase inhibitor (1.8 U/µL)) was then added and the samples heated at 37°C for 2 min. 1 µL of M-MLV RT enzyme was then added per sample and they were heated at 25°C for 10 min, 37°C for 50 min and 70°C for 15 min. Real-time PCR was performed on a ABI 7500 HT with human beta actin (Forward: 5′-CCA CCC CAC TTC TCT CTA AGG A-3′ Reverse: 5′-AAT TTA CAC GAA AGC AAT GCT ATC A-3′) as the endogenous house keeping gene and the specific GFP primers (Forward: 5′-GGG CAC AAG CTG GAG TAC AAC-3′, Reverse: 5′-CAC CTT GAT GCC GTT CTT CTG -3′). 7.5 µL of master mix containing 5 µL of 2X SYBR Green master mix (MAF Centre, U. of Alberta) and 2.5 µL primer (3.2 µM; per sample) was added to each well. Then, 2.5 µL of template of each sample was added in triplicate. A template concentration (9.76 ng/uL) was determined optimal based on a standard curve. To ensure that the efficiencies of the human beta actin and GFP primers were approximately equal, to validate use of the 2^−ΔΔCT^ method, ΔCT vs. cDNA dilution was plotted and the slope was verified to be approximately zero. Analysis was performed by 2^−ΔΔCT^ method [39] using the no-treatment group as the calibrator. Finally, the change in mRNA levels (in percent form) was calculated as follows: [% mRNA rel. NT of cells treated with CsiRNA/polymer complexes] – [% mRNA rel. NT of cells treated with GFPsiRNA/polymer complexes]. Standard deviation was calculated from the biological replicates.

### 2.9 CXCR4 Silencing in THP-1 Cells

THP-1 cells were treated with CXCR4 siRNA or control siRNA by using the polymer complexes (4∶1) as described above. At day 2 and day 3, cells were stained with 4 µL of PE-labeled mouse anti-human CXCR4 (CD184) or PE-labeled mouse IgG isotype control (BD Pharmingen) antibody in 90 µL of medium (after centrifugation and resuspension) for 45 min at room temperature. They were subsequently re-suspended in HBSS and fixed in 3.7% formalin and immediately analysed by flow cytometry (FL2 channel). As in GFP analysis, changes in mean CXCR4 levels (based on Ab fluorescence levels) and the CXCR4-positive cell population were calculated. The cell population stained with non-specific antibody was used for flow cytometry calibration (i.e., 1% CXCR4-positive population).

**Figure 1:**
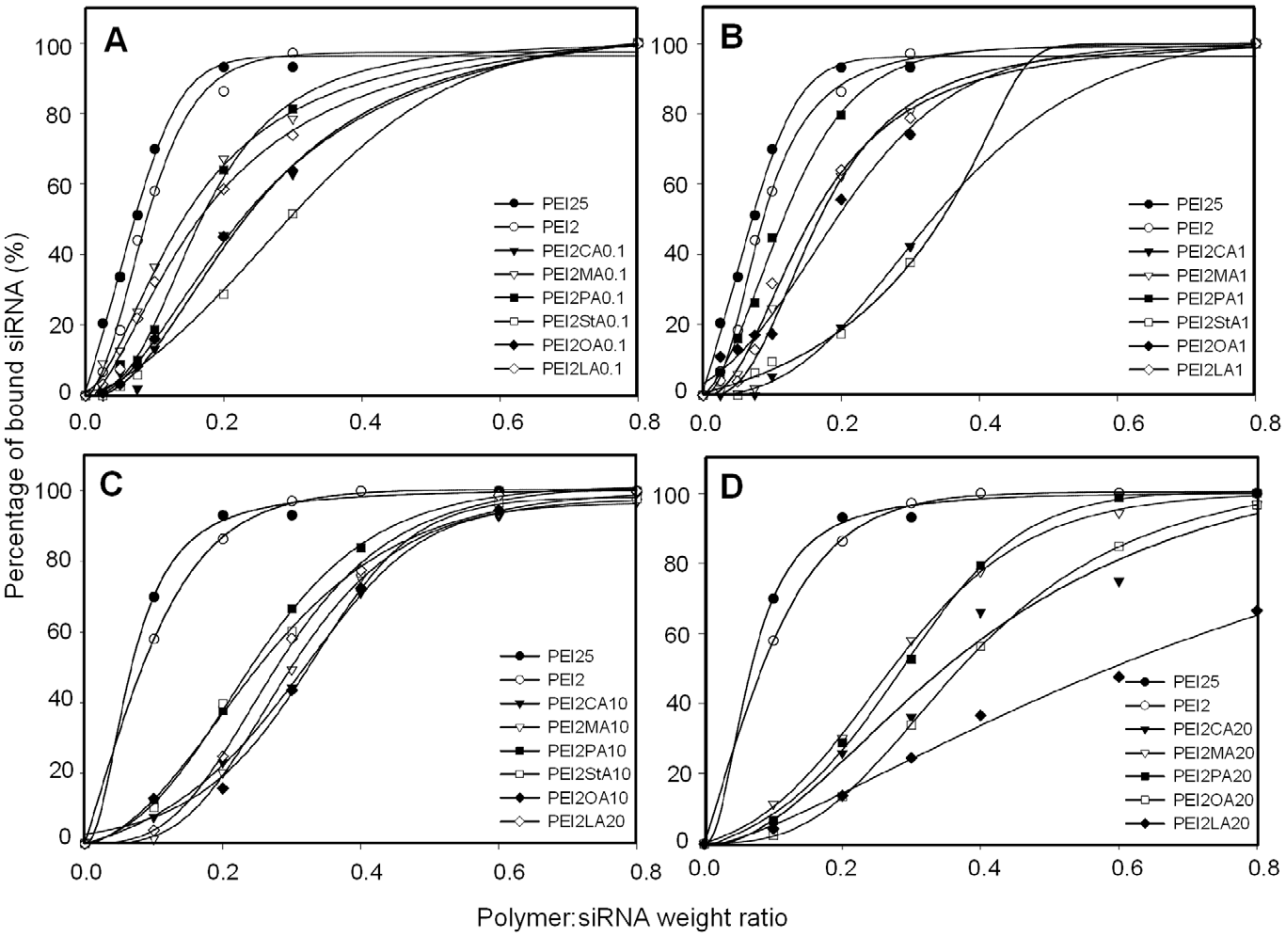
Binding of lipid-substituted polymers to siRNA. Percentage of siRNA bound as a function of polymer:siRNA weight ratio in EMSA analysis. Polymers obtained from lipid:polymer feed ratios of 0.012, 0.066, 0.1 and 0.2 are shown in **A**, **B**, **C** and **D**, respectively.

**Figure 2:**
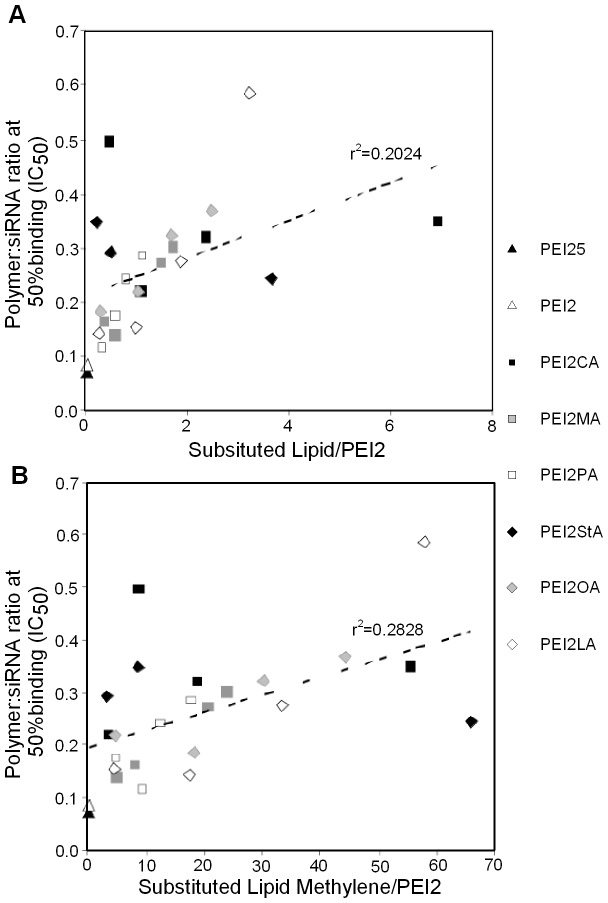
Correlations between polymer binding affinity IC_50_ and extent of lipid substitutions. IC_50_ is shown as a function of number of lipids substituted (**A**) or number of lipid methylenes substituted (**B**). As lipid substitution is increased, the binding affinity (given by IC_50_) decreased. Statistical analysis was determined by Student’s *t*-test (p<0.05).

### 2.10 Statistical Analysis

Results are displayed as the mean ± standard deviation (SD) of triplicate samples. For binding and dissociation studies, variations between the group means were analyzed as described in figures. To determine linearity, linear regression was performed; r^2^ coefficient and P values (to test for significant slope) were reported. Statistical analysis was performed with GraphPad InStat v3.06 (GraphPad Software, San Diego, CA USA).

## Results and Discussion

Lipid substitution to PEI2 was explored as a means to improve siRNA delivery to AML cells, THP-1, KG-1 and HL-60. The substituted lipids included CA, MA, PA, StA, OA and LA (in the order of increasing carbon chain length form C8 to C18) at a range of substitution levels (**Table S1**) [37], [38]. There was a general increase in lipid substitution as the lipid:PEI feed ratio was increased during the synthesis (determined by ^1^H-NMR). The highest number of lipids substituted was achieved with CA at lipid:PEI amine ratio of 0.2 (6.9 CAs/PEI). All polymers remained water soluble, except PEI2-StA20 that had the highest number of lipid methylenes substituted per PEI2 chain (89.0) and it was excluded from the study.

### 3.1 Polymer Binding to siRNA

It is imperative for the polymers to bind and neutralize the anionic charge of siRNA to form a siRNA complex. The siRNA binding ability polymers was determined by the semi-quantitative EMSA using CsiRNA. The fraction of unbounded siRNA (i.e., free siRNA capable of moving into the gel) was determined in this assay, which was used to calculate the amount of siRNA participating in complex formation. This method is similar to quantitative dye binding assay based on SYBR Green [25], but actually measures complex formation directly rather than binding of a fluorescent probe to free sites on siRNA. As expected, increasing the polymer:siRNA ratio during complex formation resulted in an increase in siRNA binding for all polymers (**Figure 1**). The binding curves typically followed a sigmoidal curve for most polymers, except a few linear curves obtained for some polymers (e.g., PEI2-LA20 in **Figure 1D**). The linear curves were usually the case for polymers with lower capacity for siRNA binding. The PEI25 and PEI2 typically yielded the most binding at a given polymer:siRNA ratio as compared to lipid substituted equivalents, indicating a lowering of binding efficiency after lipid substitutions. Based on the generated curve fits, BC_50_ values were determined as a relative measure of the siRNA binding efficiency. The PEI2 and PEI25 had the lowest BC_50_ values among the polymers (0.07 and 0.09, respectively), and all lipid-substituted polymers displayed a BC_50_ value higher than the native PEIs (**Figure 2**). For some lipids (PA and OA), a general trend of increasing BC_50_ with increasing lipid substitution was clearly evident, but not all lipids (CA and StA) gave such a clear trend. A more general relationship between the degree of lipid substitution and BC_50_ values was explored based on the correlation coefficient between BC_50_ and the extent of lipid substitution for all polymers. The obtained linear regression coefficient (r^2^∼0.2024; dashed line in **Figure 2A**) indicated a relatively weak but a significant correlation (p<0.05) between the two variables. Since each type of lipid contained a differing number of lipid carbons, we also explored a correlation between the BC_50_ and the extent of lipid Cs substituted (see **Table S1** for exact values of lipid Cs). The regression coefficient obtained was relatively higher (r^2^∼0.2828; dashed line in **Figure 2B**), again indicating a significant correlation (p<0.01) between these two variables.

**Figure 3:**
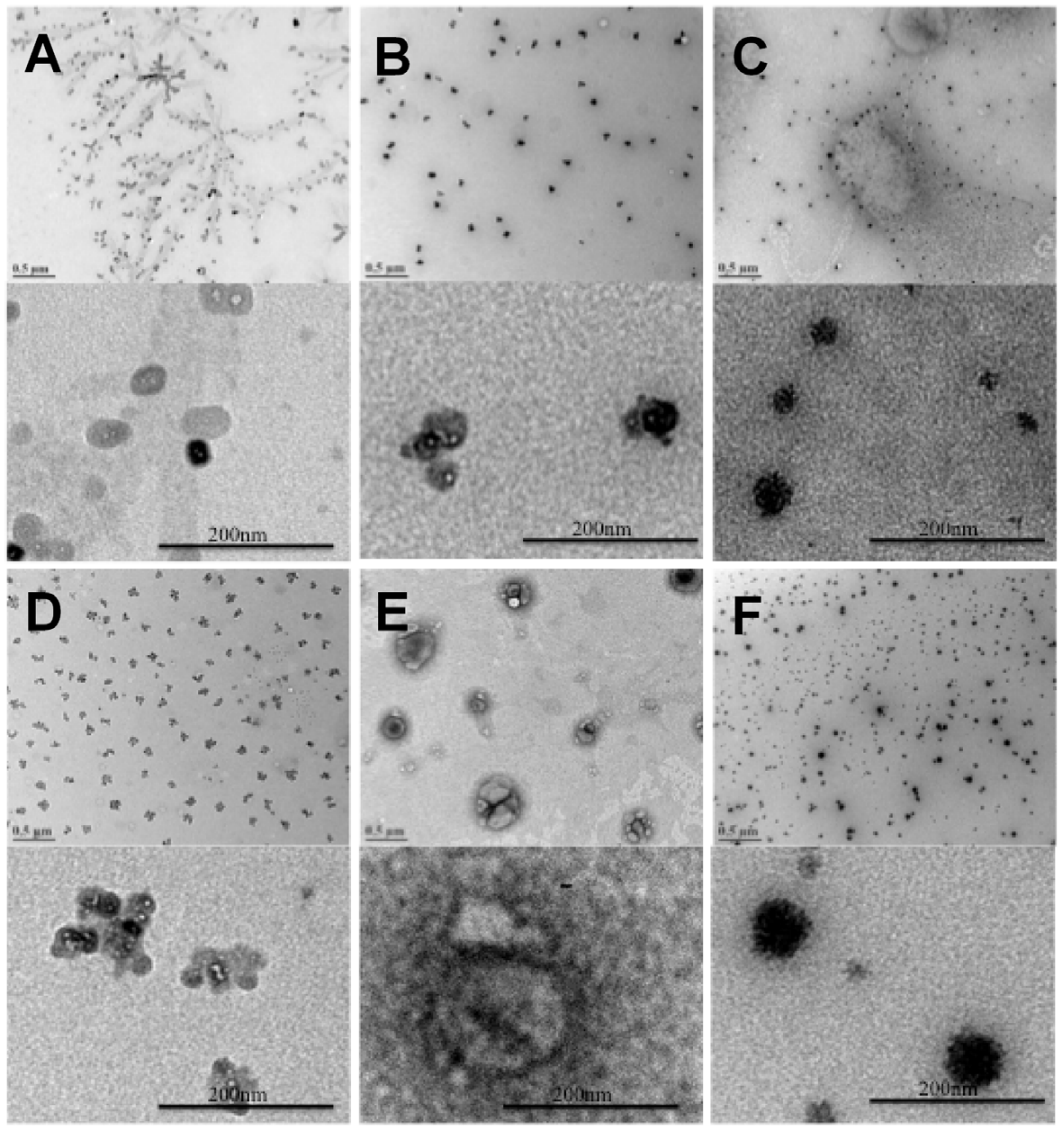
Morphology of polymer/siRNA complexes imaged by TEM. (**A**) PEI25 complexes, (**B**) PEI2 complexes, (**C**) PEI2-CA20 complexes, (**D**) PEI2-PA20 complexes, (**E**) PEI2-OA20 complexes, (**F**) PEI2-LA20 complexes. All complexes were prepared at an 8∶1 polymer:siRNA weight ratio. Scale bar in the high magnification images indicates 200 nm.

TEM imaging for the complexes with native PEIs (PEI25 and PEI2) and representative CA, PA, OA and LA substituted PEI2 are summarized in **Figure 3**. Distinct complexes were observed in most cases, but some polymers (PEI2 and PEI2-PA) gave aggregated particles where smaller spherical particles appeared to fuse together. Fusing of particles in TEM images have been seen in other studies as well [40], which was likely due to drying during the sample preparation. Most complexes appeared relatively homogenous (similar contrast throughout the complex) with the exception of PEI2-OA, where spherical particles appeared to be multiphasic. The size of individual complexes were typically <100 nm, with PEI2-OA particles being notably larger (>200 nm). Directly comparable images of TEM complexes, such as PEI25/siRNA or PEI2/siRNA complexes, are not available in the literature; however, TEM imaging of PEI25/plasmid DNA complexes were reported to be larger than our PEI25/siRNA complexes [41], consistent with larger size of plasmid DNAs used to assemble the particles.

**Figure 4:**
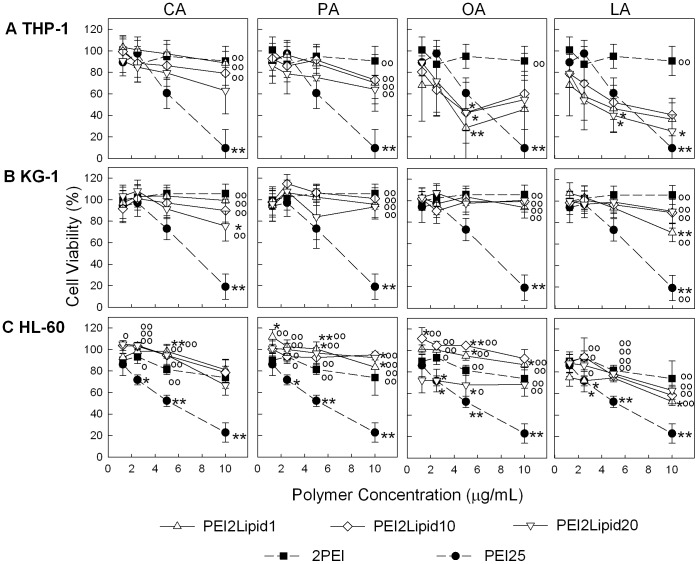
Cytotoxicity of complexes on THP-1, KG-1 and HL-60 cells (top, middle and bottom panel, respectively). Cell viability values are expressed relative to no-treatment control. Viability was measured 24 h after incubation of complexes with cell lines. PEI25 displays a linear relationship with increased cytotoxicity in line with increasing concentration. Effect of lipid-substituted polymers on cell viability was similar to that of unmodified PEI2. * : *p*<0.05, ** : *p*<0.01, as compared to PEI2 and ^o^ : *p*<0.05, ^oo^ : *p*<0.01 as compared to PEI25, using one-way ANOVA tests with Dunnett post test.

In order to minimize the scale of further experiments, the lowest substitution for each polymer was excluded from the experiments as they are expected to be the least different from the unmodified PEI2. Additionally, MA- and StA-substituted polymers were excluded, as these substitutes did not appear to be unique in the extent of substitutions and the siRNA binding studies.

### 3.2 Cytotoxicity of Polymeric Carriers

It is well established that the high MW PEI25 generally displays high cytotoxicity in contact with cells, whereas low MW PEIs display minimal cytotoxic effects [29]–[34]. High MW polymers were suggested to be more effective in creating membrane invigilations and/or pores, which is desirable for siRNA delivery, but this also causes more harm on the cells by disrupting membrane integrity [42]. While lipid-substitution is intended to increase membrane affinity of polymers, our lipid-modified polymers are expected to expose the cells to lipid concentrations of 1–10 µM, assuming a polymer concentration of ∼6 µg/mL in contact with the cells (practical concentration used for siRNA delivery) and average lipid substitutions of ∼3 lipids/PEI2. The lipids are naturally occurring molecules and palmitic, oleic and lineloic acids are present in plasma membranes. Additionally, the toxicity of lipids on leukemia cell lines have been investigated; tolerable concentration of lipids in Jurkat (human T leukemic) and Raji (human B leukemic) cell lines depended on the specific lipids, but all concerned lipids were tolerable at ∼50 µM [43], a value much higher than lipid concentrations to be exposed to cells with our carriers.

The cytotoxicity of the complexes on AML cells is summarized in **Figure 4**. As expected, PEI25 displayed an obvious, concentration-dependent toxicity in all cells. The PEI2 displayed minimal toxicity that was evident only in HL-60 cells (see **Table S2** for detailed analysis of cytotoxicity trends). For lipid-substituted PEI2, a concentration dependent loss of viability was evident for some cell-polymer pairs, but other polymers did not display toxicity in the investigated concentration range. However, cytotoxicity of the PEI2 and PEI2-lipids did vary among the cell lines. For THP-1 cells, minimal toxicity was observed with CA and PA substitutions, while OA and LA substituted PEI2 gave a significant decrease in cell viability. In KG-1 cells, minimal, if any, decrease in cell viability was seen with CA, PA and OA substitutions, while a slight increase in cytotoxicity was seen with LA substituted PEI2. For HL-60 cells, PEI2 substitutions with CA and PA displayed no changes in cytotoxicity, but OA and LA substitutions displayed a small negative effect on cell viability. Taken together, LA was the only lipid substituent that clearly increased the cytotoxicity of the polymers in all three cell lines. This was presumably due to better interaction of this type of polymer with these AML cells.

Since a major concern of polymeric carriers is the dose-dependent cytotoxicity [44], as obviously manifested with the PEI25, it is notable that our carriers did not display definitive dose-response curves in investigated cell lines. Relatively low cytotoxicity is the likely reason for the lack of clear dose-response curve. We previously noted that lipid substitution generally increased the toxicity of PEI2 on anchorage dependent bone marrow stromal cells [38] and breast cancer cell line MDA-435 [25]. To further explore this issue with leukemic cells, a correlation between the lipid substitution and the resulting cytotoxicity was explored as in the binding studies (**Table S3**). Very few obvious correlations occurred in this analysis; the strongest trends were seen at the highest polymer concentrations of 10 µg/ml where high r^2^ and significant slopes (p values) were observed for CA- and PA-substitutions in KG-1 cell lines. Again, relatively low cytotoxicity in the working range did not allow for a strong correlation and toxicity at higher concentrations was not explored since this is not the practical range for siRNA delivery.

**Figure 5:**
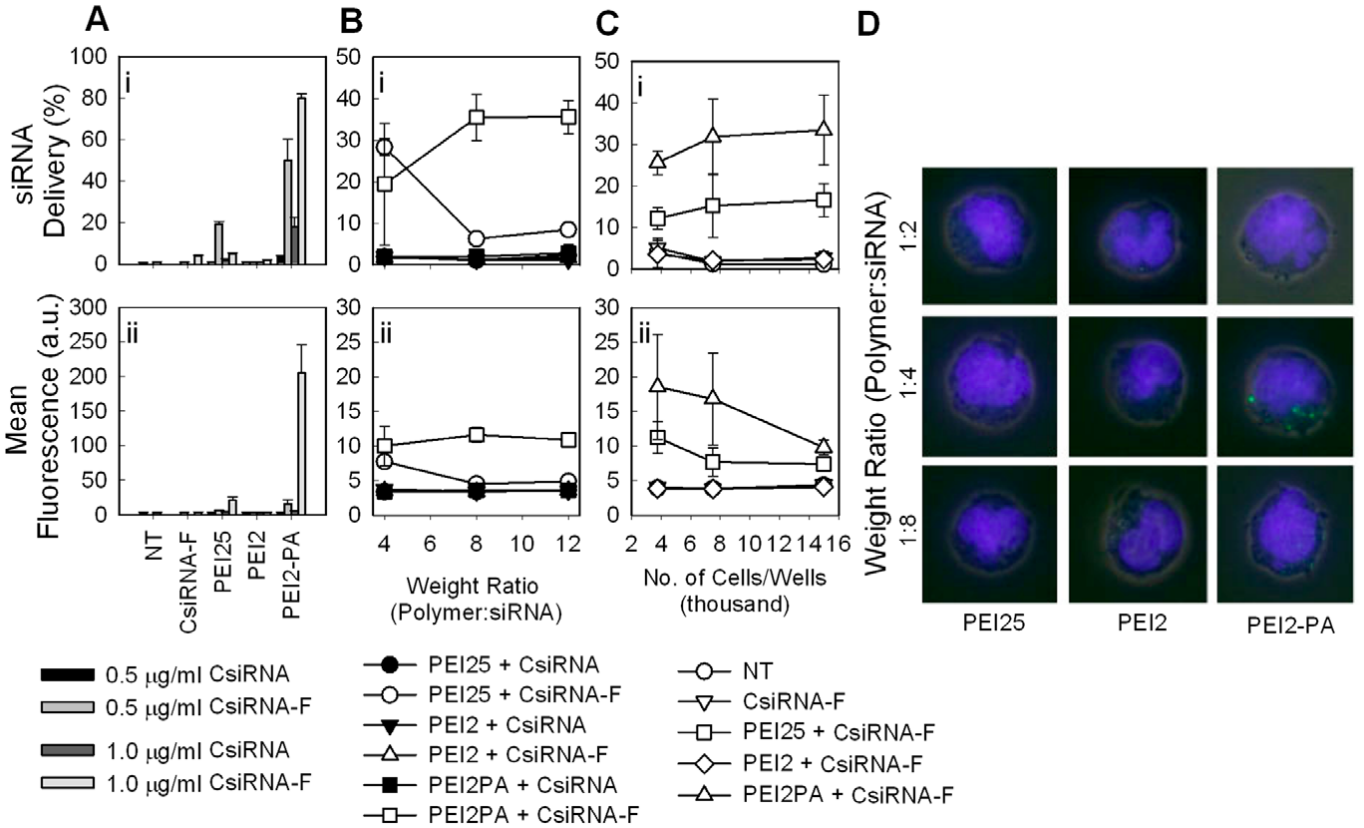
siRNA delivery to THP-1 cells. (**A**) varying complex dose (0.5 and 1.0 µg/mL siRNA). (**B**) varying polymer:siRNA weight ratio. (**C**) varying initial cell number (0.35 µg/mL siRNA). The results are summarized as (i) percentage of siRNA-associated cells in cell population, and (ii) mean fluorescence of cells due to complex association. (**D**) confocal microscope images of individual cells (0.5 µg/mL siRNA). Hoechst stained nucleus in blue and FAM-labelled siRNA-polymer complexes in green. Polymer:siRNA ratios were 2∶1, 4∶1 and 8∶1 from top to bottom panel.

### 3.3 siRNA Delivery with PA-substituted PEI2 to THP-1 Cells

Initial siRNA delivery studies were performed with THP-1 cells and by using PEI2-PA as a prototypical carrier. Two concentrations of siRNA complexes (corresponding to 36 and 72 nM siRNA and, 4 and 8 µg/mL polymer) were used in this study, as well as an unlabeled siRNA (CsiRNA) as a control to account for the possibility of autofluorescence displayed with certain carriers [45]. The percentage of cells with siRNA uptake and the mean siRNA fluorescence in cells are summarized in **Figure 5A**. Exposure of the cells to CsiRNA did not indicate significant autofluorescence at the low concentration, but a significant autofluorescence was evident at the high polymer concentration (8 µg/mL) with PEI2-PA. The PEI2-PA was successful in delivering siRNA to majority of the cells (>50%) at both doses and also demonstrated higher delivery than PEI25, which gave a lower percentage of siRNA-positive cells (<16%) that decreased at higher complex dose. This decrease was likely due to high toxicity of PEI25 at the 8 µg/mL used in this experiment. The low effectiveness of PEI25 was unlike most siRNA delivery studies reported in the literature that typically employed anchorage-dependent cells, such as human breast cancer cells [25], [35], [46], mouse albino neuroblastoma cells [27], human ovary cells [28], human prostate carcinoma cells [46], human cervical cancer cells [26], and mouse glioblastoma cells [47]. The ineffectiveness of PEI25 in haematopoietic cell lines was previously noted for delivery of plasmid DNA [48], which found PEI2 to be superior to PEI25. Unlike the study on the plasmid DNA delivery, the PEI2 was not effective in our hands for siRNA delivery.

**Figure 6:**
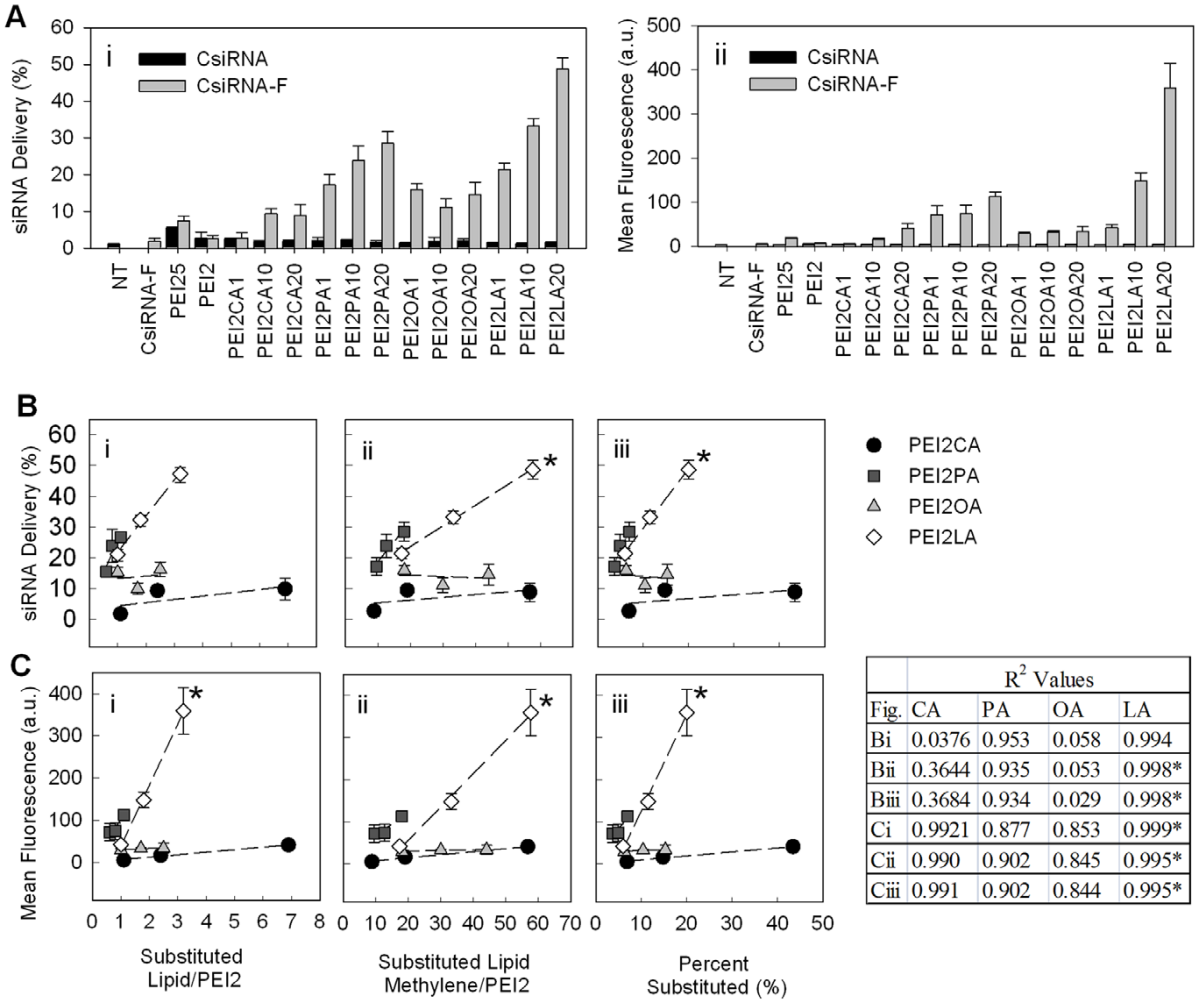
Effect of lipid substitution on siRNA delivery to THP-1 cells. Polymer:siRNA ratio was 8∶1 and siRNA concentration was 25 nM (0.35 µg/mL). (**A**) siRNA delivery percentage (percentage of cells with complexes, i) and mean fluorescence (mean fluorescence of cells due to fluorescence labelled siRNA-polymer complexes, ii). (**B**) Correlations between siRNA delivery percentage and lipid substitution. (**C**) Correlations between mean fluorescence and lipid substitution. Very strong positive correlations (r^2^ values) are seen with PA and LA regardless how the lipid substitution is expressed (no of lipid per PEI2, no of lipid methyl carbons per PEI2 or percentage of PEI2 amines substituted) and with both siRNA delivery and mean fluorescence. Strong correlation (r^2^ value) is seen with CA when considering mean fluorescence. * indicates where the slope is statistically significant.

To further compare the relative efficiency of PEI25, PEI2 and PEI2-PA, siRNA delivery was explored as a function of polymer:siRNA ratio (**Figure 5B**) and seeded cell density (**Figure 5C**). As before, PEI2 was not effective under all investigated conditions. While PEI25 was more effective at lower ratio (4∶1), PEI2-PA was more effective at higher ratios (8∶1 and 12∶1), indicating the polymer:siRNA ratio to be critical for uptake. As the cell concentration was increased, the percentage of siRNA-associated cells remained the same, but the mean fluorescence/cell was decreased (**Figure 5C**), indicating less siRNA uptake/cell at higher cell concentrations. Finally, confocal microscopic analysis of the siRNA uptake confirmed the quantitative results obtained. Distinct cell-associated complexes were clearly seen with PEI2-PA, but not with PEI25 and PEI2 (**Figure 5D**). It was therefore clear that the lipid substitution on PEI2 (PA in this case) mediated improved delivery of siRNA to the leukemic cells.

**Figure 7:**
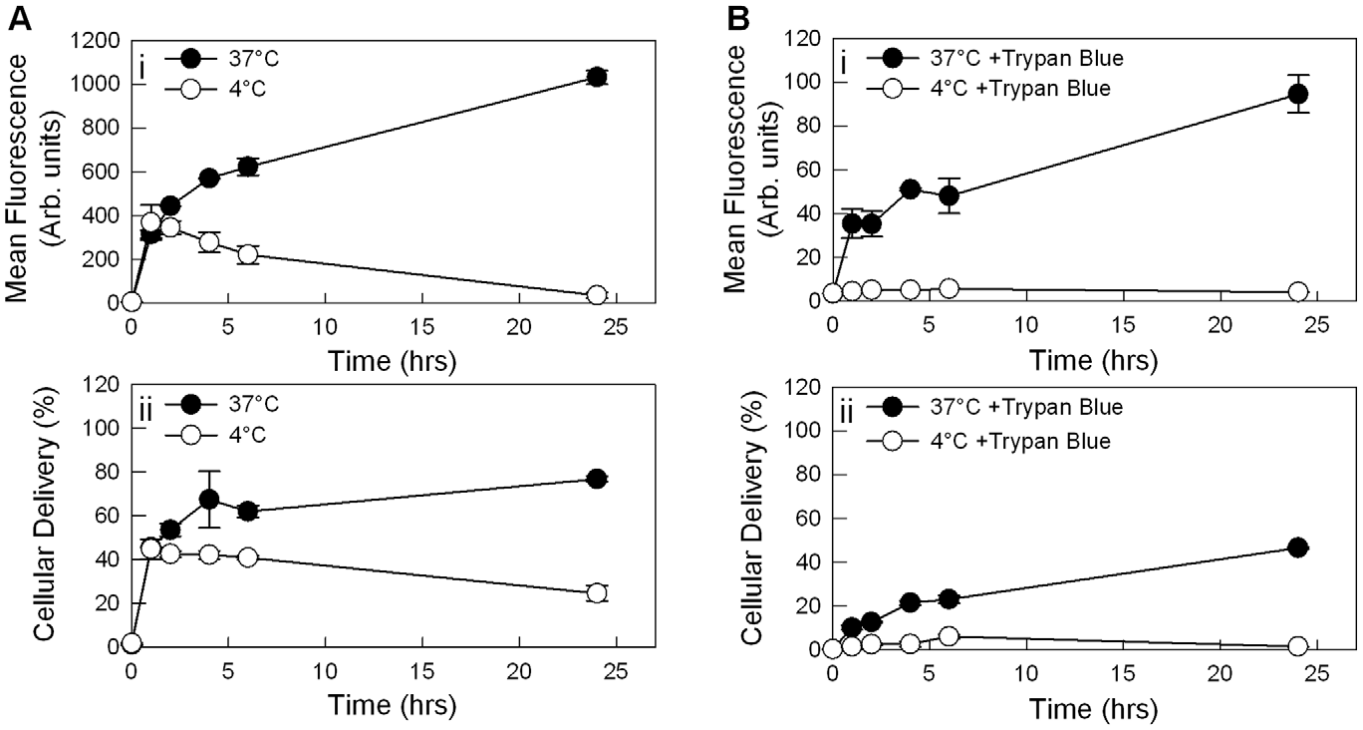
Effect of temperature (4 and 37°C) and trypan blue treatment on siRNA delivery to THP-1 cells. PEI2-LA (2.1 LA/PEI2) was used in this study with polymer:siRNA ratio of 8∶1 and final siRNA concentration of 25 nM (0.35 µg/mL). (**A**) siRNA delivery based on mean FAM fluorescence (i) and FAM-siRNA positive cell population (ii) with untreated cells (**B**) siRNA delivery based on mean FAM fluorescence (i) and FAM-siRNA positive cell population (ii) with cells treated with trypan blue.

### 3.4 Effect of Lipid Substitution on siRNA Delivery

To develop a broader understanding of the role lipid substitution on siRNA delivery, PEI2 substituted with CA, PA, OA and LA were used to evaluate siRNA delivery to THP-1 cells (**Figure 6**). The siRNA delivery varied significantly among the polymers, whether it was assessed by the siRNA-positive cell population (**Figure 6Ai**) or the mean siRNA level per cell (**Figure 6Aii**). Based on these two parameters, siRNA delivery was correlated to the number of lipids substituted/PEI2 (**Figure 6Bi** and **6Ci**), the number of lipid methylenes/PEI2 (**Figure 6Bii** and **6Cii**) and percentage of lipid substitution (**Figure 6Biii** and **6Ciii**). As shown in the table in **Figure 6**, strong correlations (see r^2^ values listed in the figures) were observed with PA and LA substitution in all cases. These positive correlations were indicative of lipid substitutions to be directly responsible for intracellular siRNA delivery. Among the polymers, PEI2-LA polymers appeared to be most effective, based on the strong correlations between siRNA delivery and LA substitutions, as well as absolute levels of siRNA delivery per cell. It was also clear that the enhanced delivery was dependent on the individual lipid, as the explored correlations failed if all lipid-substituted polymers were considered together. This was unlike the case with the binding studies where the correlation was valid with all lipids, indicating a similar role of lipids on the siRNA binding, but significantly different roles in delivering the siRNA to the cells.

**Figure 8:**
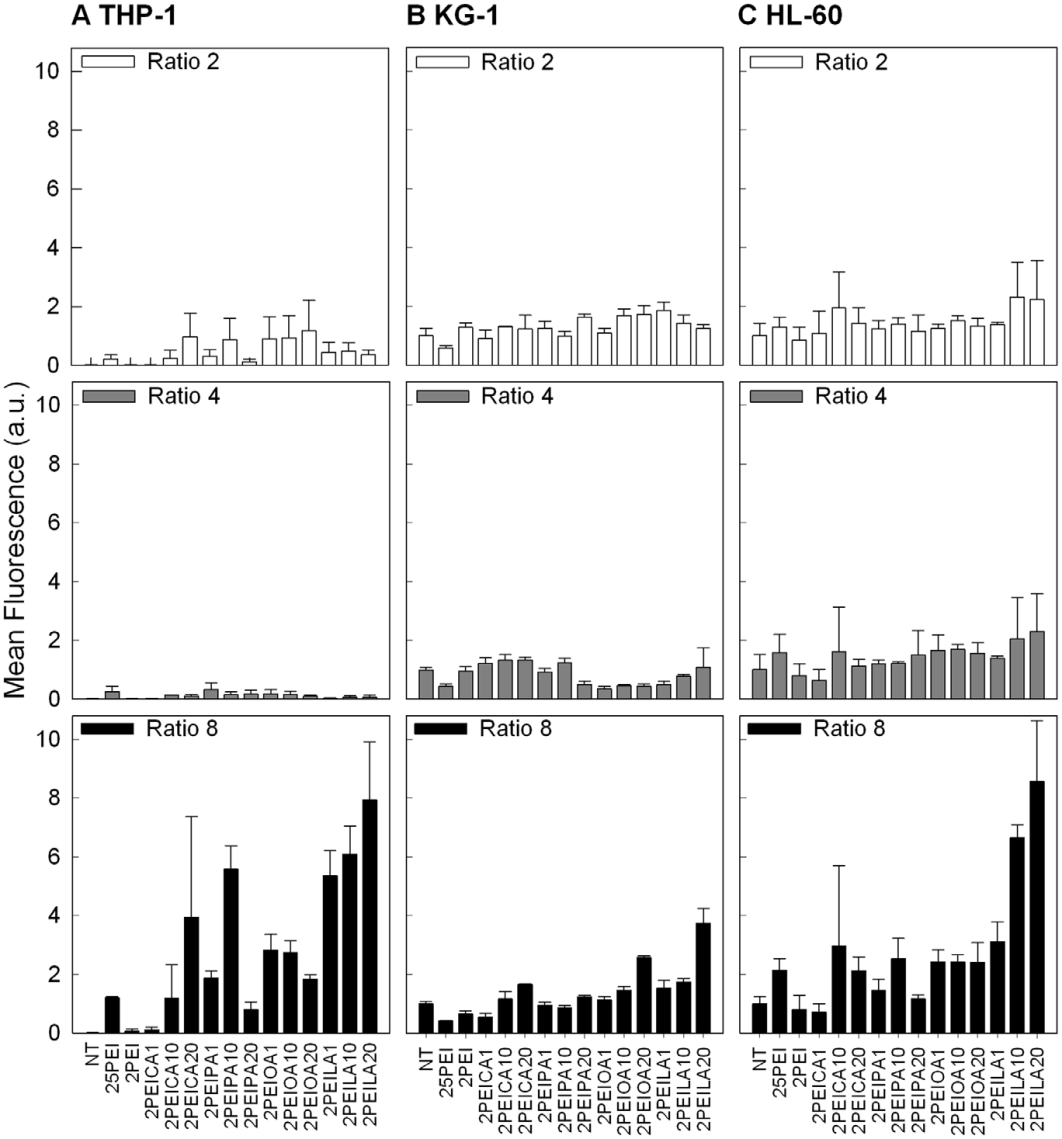
siRNA delivery to THP-1, KG-1 and HL-60 cells at various polymer:siRNA weight ratios. (**A**) THP-1 (**B**) KG-1, and (**C**) HL-60 cells. Fluorescence intensity refers to the mean fluorescence of the cell population. Non-labeled control siRNAs did not show any significant fluorescence (autofluorescence) and were removed for figure clarity. siRNA concentration was 25 nM (0.35 µg/mL) and polymer:siRNA weight ratios were 2∶1 (top panel), 4∶1 (middle panel) and 8∶1 (bottom panel).

To determine the extent at which the complexes are internalized as opposed to remaining surface-bound, a time course of complex uptake was performed (**Figure 7**). The polymer that provided the highest siRNA delivery, PEI2LA, was used for this purpose in THP-1 cells. Trypan blue staining was employed to differentiate between surface bound and internalized complexes, since it quenches the fluorescence of surface-bound complexes [49], as well as incubation at 4°C since it prevents active complex internalization. A gradual increase in cell-associated siRNA was evident in the first 24 hours for the cells incubated at 37°C (**Figure 7A**), where ∼80% of the cells became siRNA-positive (**Figure 7Aii**). For cells incubated at 4°C, no further increase in cell-associated siRNA was evident after the initial binding at 1 hour (**Figure 7A**). With trypan blue quenching, a continuous increase in siRNA uptake was evident at 37°C, but not at 4°C (**Figure 7B**). This was consistent with abolished active uptake at the latter temperature. Whereas the proportion of siRNA-positive cells gradually increased to ∼42% at 37°C, this value remained <7% for 4°C incubated cells during the 24-hour study period (**Figure 7Bii**). We note that the fluorescence levels obtained by trypan blue treatment was greatly diminished (**Figure 7Bi**), consistent with results reported by an independent group [49]. As trypan blue coats the surface of the cells, it is likely that it decreased the excitation of the internalized fluorescent complexes within the cells.

Although the beneficial effect of lipid modification of carriers in cellular delivery of nucleic acids is established [50], the mechanism(s) by which they due so remains ill-defined. It has been suggested that the lipid modifications may elicit specific biological responses in interacting with cellular membrane, facilitating uptake and intracellular transport [50]. From a physical perspective, membrane phospholipids, consisting of various combinations of lipids, significantly contribute to the membrane stability, permeability and fluidity. Saturated lipids such as CA (C8) and PA (C16) are linear and allow tighter membrane packing leading to decreased fluidity and permeability, whereas unsaturated lipids (one double bond in OA (C18∶1) and two double bonds in LA (C18∶2)) introduces non-linear chains and disorder (and fluidity) into membranes [51]. Therefore, it is not surprising that the LA-substituted complexes will increase the membrane fluidity the most and display the highest uptake. The composition of lipids in the cellular membrane is another possible cause of variations in delivery. In an analysis of lipid composition in AML cells, the weight percentage of PA, OA and LA were 20.8±1.2%, 15.9±2.2% and 7.0±1.0% respectively (values equivalent to healthy controls [52]). Similar percentages were also reported in Jurkat (T-lymphocyte), Raji (B-lymphocyte) K562 cells, and foetal calf serum [43], [53]. LA content seems to be significantly less than the PA and OA contents. After incubation in LA-supplemented medium, the LA content of cellular membranes can be increased extensively (∼20 times) [53]. It is conceivable that lipids that are present at lower concentrations originally (i.e., LA) would be taken up and incorporated in the cellular membrane to a greater degree. Thus, in order to investigate the effect of specific lipid uptake into the cellular membrane, cells were incubated with the free LA (0–100 µM) for 24 h hours followed by siRNA uptake for 24 h. The exposure of cells to LA prior to adding the complexes did not effect siRNA delivery percentages, regardless of using polymer/siRNA complexes at 2∶1 or 8∶1 polymer:siRNA (**Figure S2A)**. Additionally, incubation of LA, OA and StA (0–50 µM) with simultaneous siRNA complex (PEI2-LA, PEI2 and PEI25) treatment did not influence the extent of siRNA delivery (**Figure S2B**). These results suggested that free lipids did not affect the uptake of the complexes, so that specific uptake was likely not the reason for increased uptake of LA-containing complexes.

**Figure 9:**
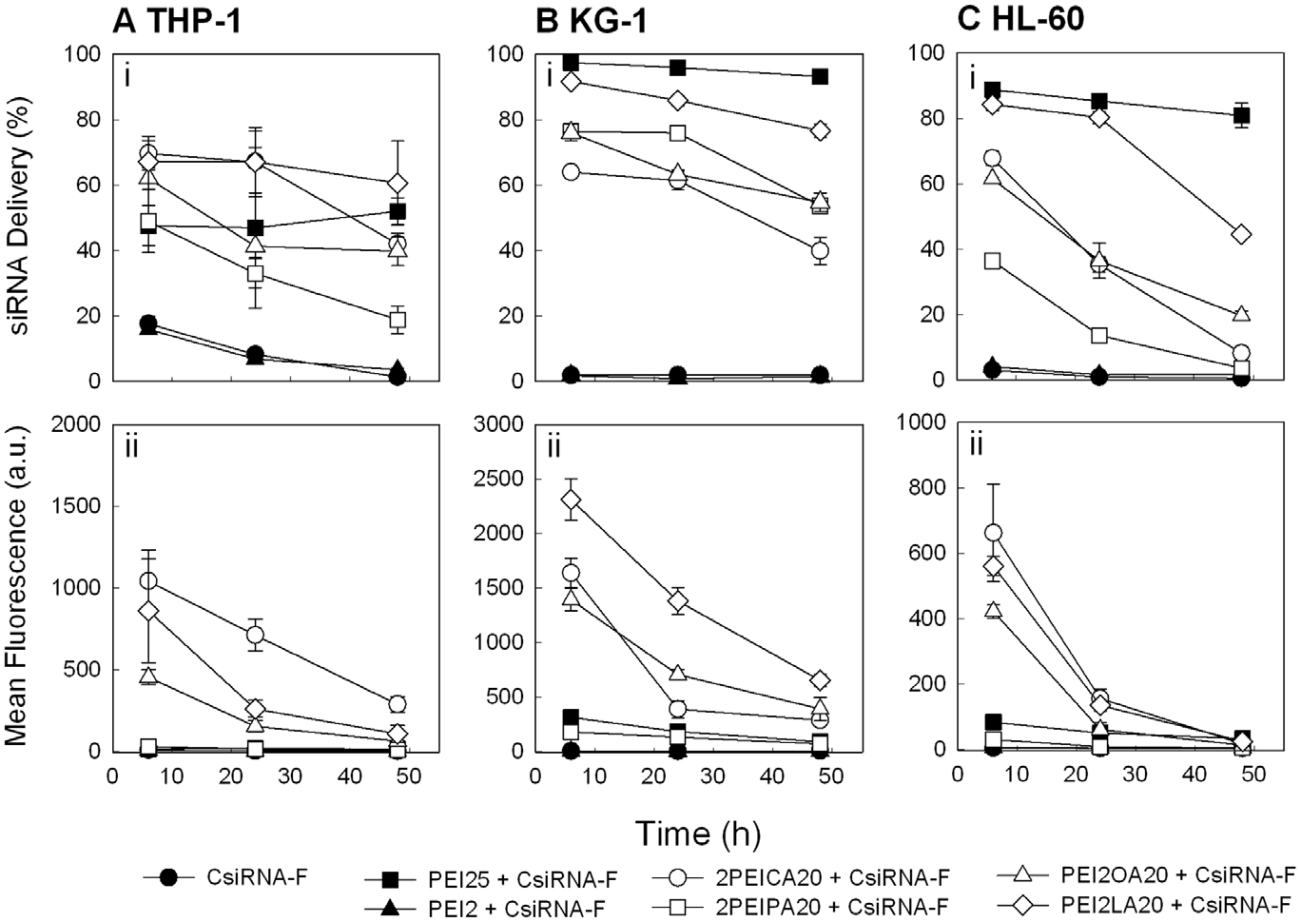
siRNA delivery to THP-1, KG-1 and HL-60 cells at various time points. (**A**) THP-1 (**B**) KG-1, and (**C**) HL-60 cells. Non-labeled control siRNAs did not show any significant fluorescence (autofluorescence) and were removed for figure clarity. siRNA concentration was 25 nM (35 µg/mL) and polymer:siRNA weight ratio was 8∶1. The results are summarized as percentage of siRNA positive cells (top panel), mean fluorescence per cell (middle panel).

**Figure 10:**
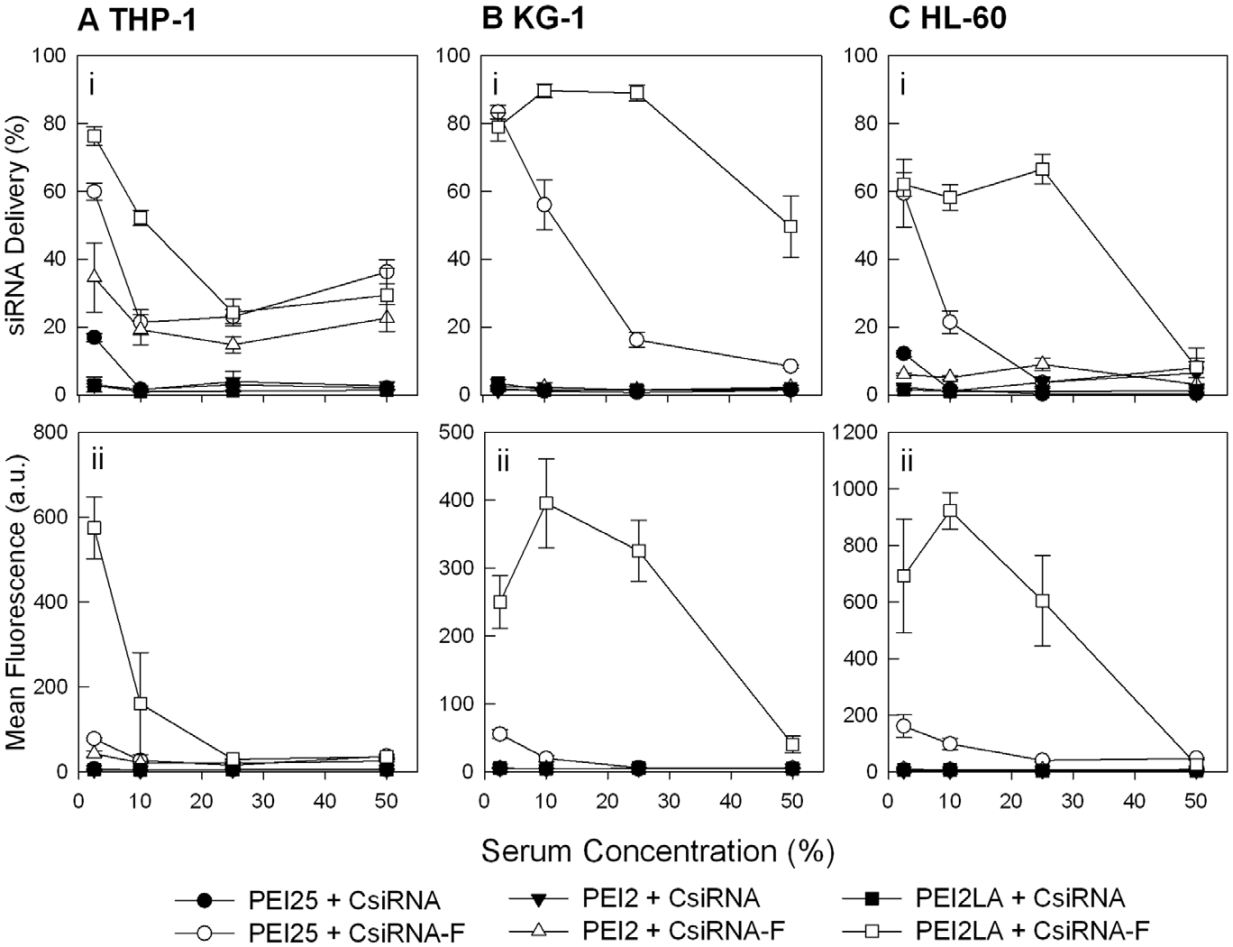
Effect of serum on siRNA/polymer complex delivery to THP-1, KG-1 and HL-60 cells. (**A**) THP-1 (**B**) KG-1, and (**C**) HL-60 cells. siRNA concentration was 25 nM (35 µg/mL) and polymer:siRNA weight ratio was 8∶1. The results are summarized as percentage of siRNA positive cells (top panel) and mean fluorescence per cell (middle panel).

**Figure 11:**
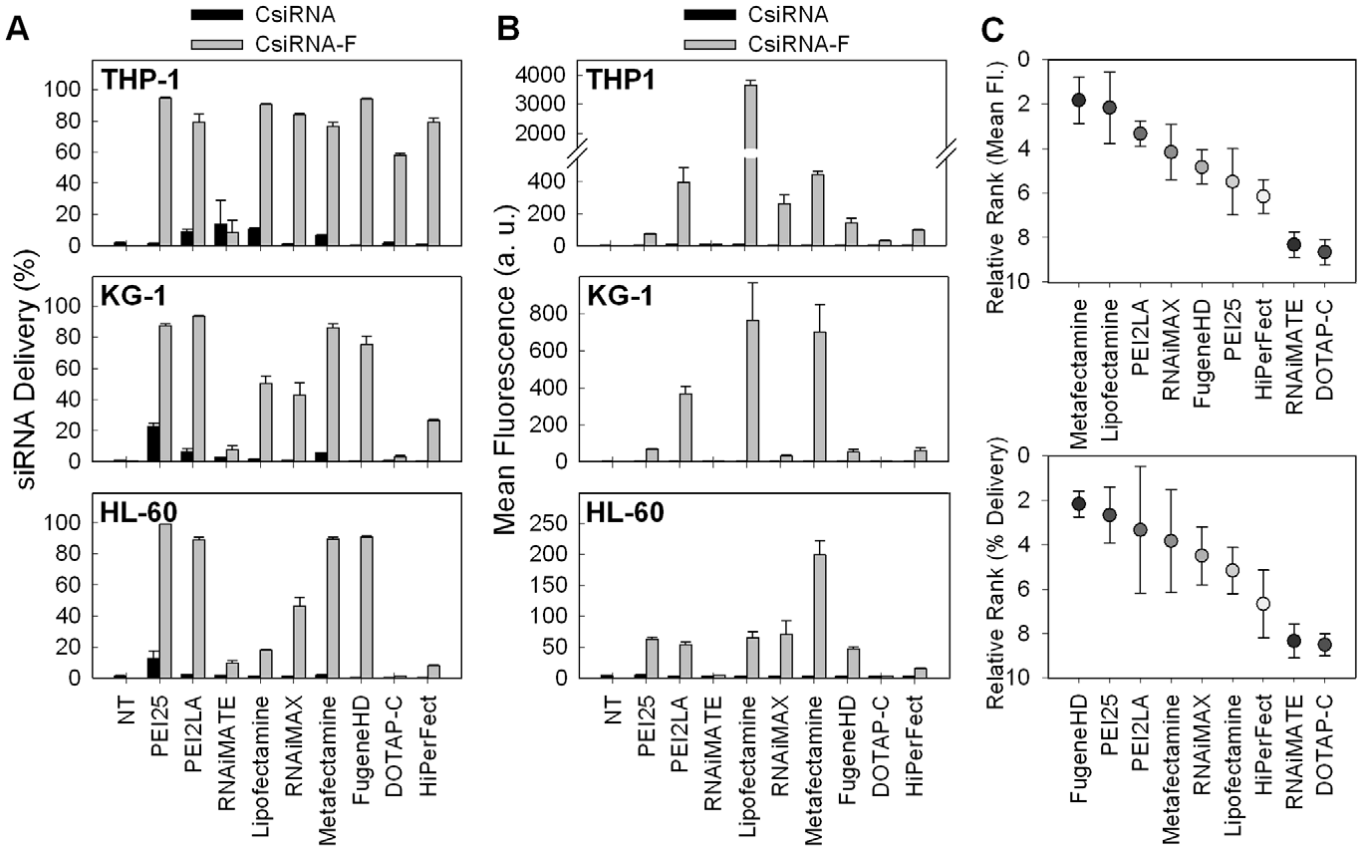
siRNA delivery to THP-1, KG-1 and HL-60 cells by commercial reagents. siRNA delivery after 24 hours was expressed as the siRNA-positive cell population (**A**) or mean fluorescence of cell population (**B**). (**C**) Relative ranking of various reagents based on mean fluorescence (top panel) or percentage of siRNA-positive cell population (bottom panel). Results from all three cell types were pooled for the ranking.

### 3.5 siRNA Delivery to Other AML cells

Two other AML cell lines, the acute myelogenouse leukemia (M1) KG-1 cells and the acute promyelocytic leukemia (M2) HL-60 cells, were next tested for siRNA delivery with the modified PEI2s, along with the THP-1 cells (**Figure 8**). Of the cell lines studied, KG-1 (M1) is the least differentiated (myeloblast), HL-60 (M2) is in the early stages of differentiation (promyeloblast) and THP-1 (M5) is the most differentiated (monocyte). As classification is dependent on differentiation stage, the cells vary in expression of the differentiation markers CD11b and CD14 (monocytic markers) and CD33, CD13, CD65s, CD15/15s (myeloid markers), as summarized in [54]. The siRNA delivery was investigated at polymer:siRNA ratios of 2∶1, 4∶1 and 8∶1. Various lipid substitutions were successful for siRNA delivery to THP-1 cells, given by the large increases in siRNA delivery after lipid substitution on PEI2 (**Figure 8A**). In the KG-1 and HL-60 cells, LA-substituted PEI2 was again the most effective (**Figure 8B and 8C**). Of the three different polymer:siRNA ratios tested, the 8∶1 ratio consistently gave the best delivery. This was attributed to higher cationic charge of the complexes formed at higher polymer:siRNA ratios [24], which should facilitate better binding to anionic cell surfaces. The PEI25 and PEI2 did not appear to be an effective delivery agent for the KG-1 and HL-60 cells either, confirming our previous observation with THP-1 cells. It was also evident that the cells displayed differing propensity to uptake polymer/siRNA complexes; whereas THP-1 appeared to be most readily display siRNA uptake, KG-1 cells displayed the least uptake. This was expected since KG-1 being the least differentiated phenotype (leukemic progenitor cell) with minimal endocytic activity and smaller size (12–16 µm) and THP-1 being the most differentiated with larger size (15–20 µm) [55]. It will be important to further explore the molecular basis of this observation, since it might be an indicative of patient-to-patient variations in siRNA delivery.

**Figure 12:**
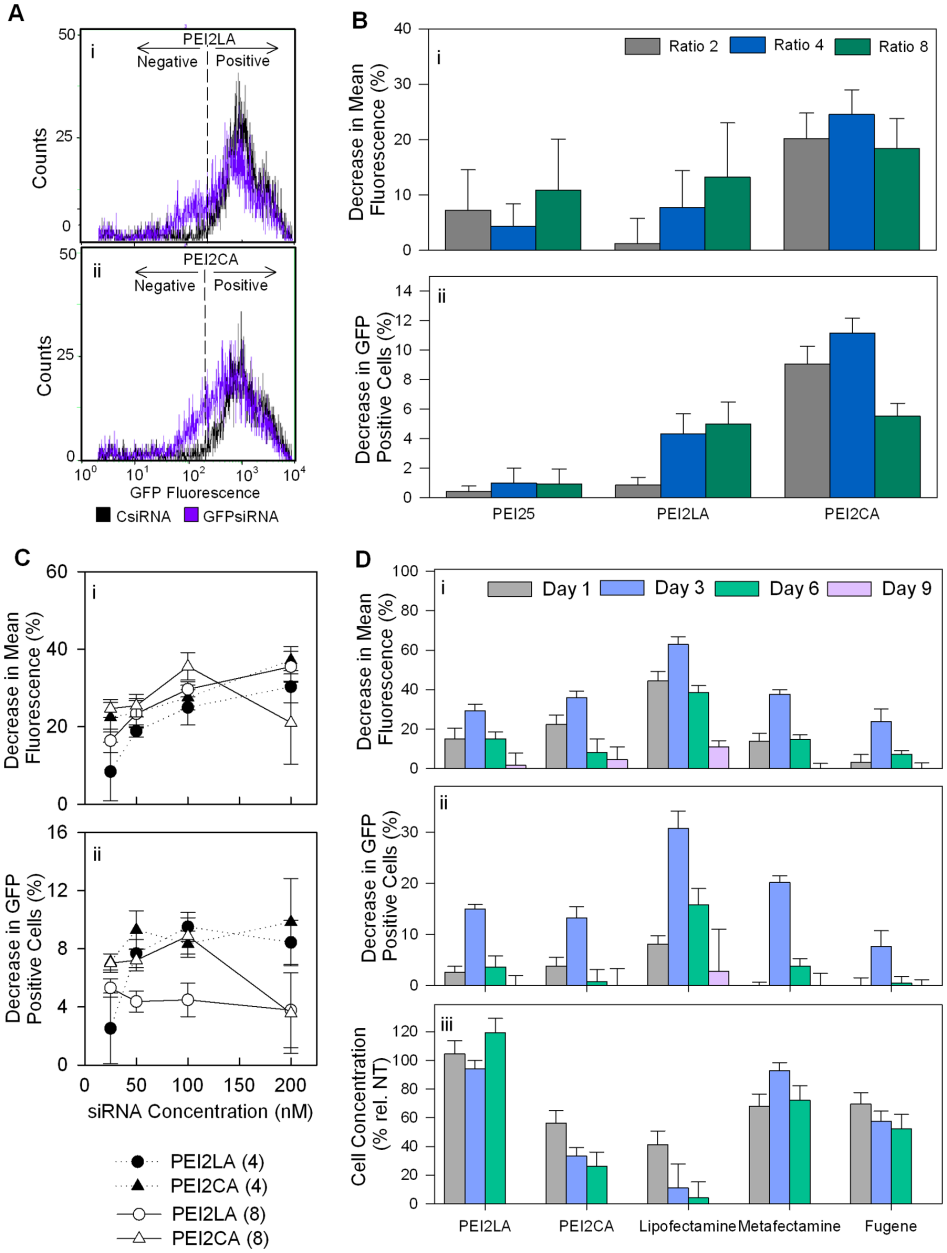
Silencing in GFP-expressing THP-1 cells. (A) The cell counts as measured by flow cytometry (expressed as a percentage of non-treated cells). (**B**) Silencing was assessed after 3 days of siRNA treatment (50 nM) and expressed as decrease in mean GFP fluorescence or decrease in GFP-positive cells. The polymer:siRNA ratios were 2∶1, 4∶1 and 8∶1. (**C**) Dose-response curves for GFP silencing between 25 and 200 nM siRNA treatment. The CA and LA substituted polymers were used at the polymer:siRNA ratios of 4∶1 and 8∶1, and silencing was assessed after 3 days of treatment. (**D**) Silencing by CA- and LA-substituted polymers and three commercial reagents (Lipofectamine™ 2000, Metafectamine and Fugene HD). The extent of silencing was summarized over a course of 9 days and expressed as decrease in mean GFP fluorescence (top panel) or decrease in GFP-positive cells (middle panel). The lipid substitutions of the polymers used were 2.1 LA/PEI (PEI2-LA) and 6.9 CA/PEI (PEI2-CA).

**Figure 13:**
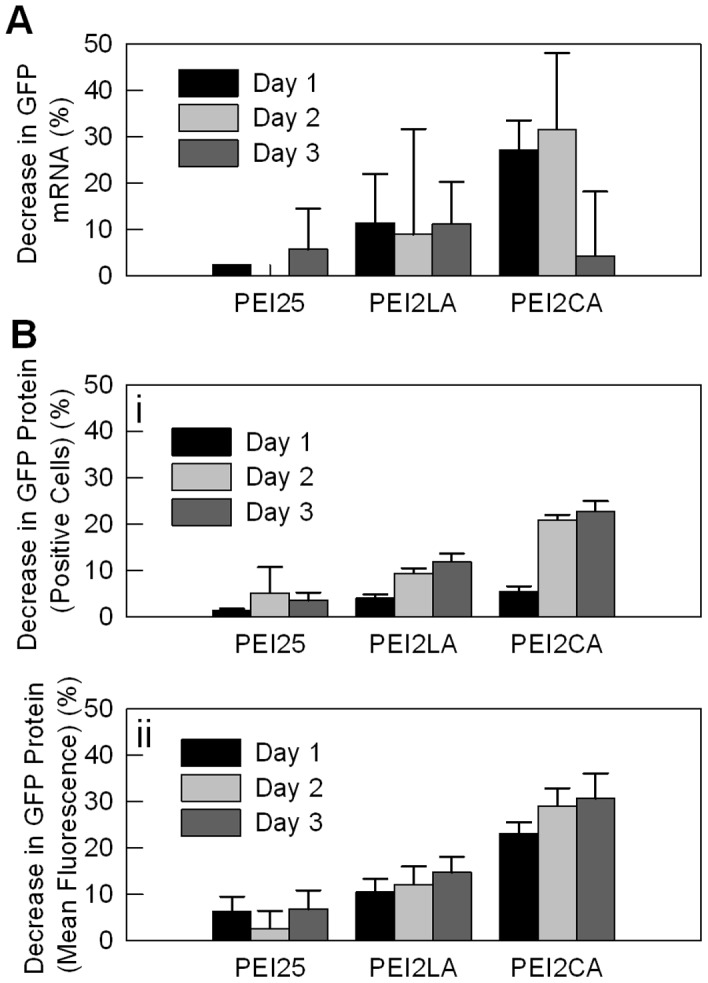
GFP mRNA and protein suppression in THP-1 cells. (**A**) Decrease in GFP mRNA levels, and (**B**) decrease in GFP protein levels (i: based on mean GFP fluorescence and ii: based on GFP-positive cell population). The GFP-positive THP-1 cells were treated with 50 nM GFP siRNA (or control siRNA) delivered with PEI25, PEI2-LA (2.1 LA/PEI) and PEI2-CA (6.9 CA/PEI; 4∶1 =  polymer:siRNA ratio) for 1 to 3 days, after which the cells were harvested for PCR (**A**) and flow cytometry (**B**).

**Figure 14:**
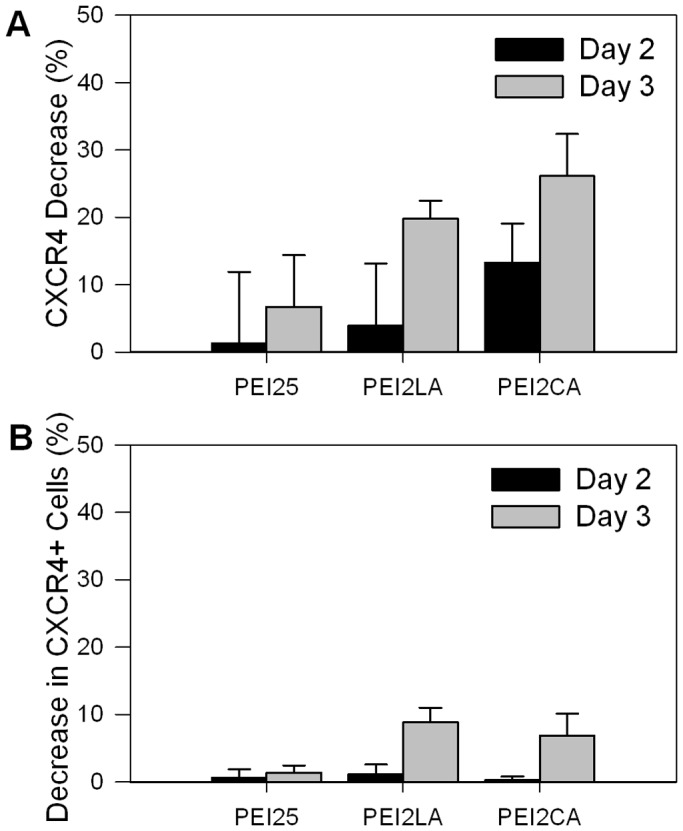
CXCR4 Silencing in THP-1 cells. Changes in CXCR4 levels based on (**A**) mean CXCR4 fluorescence intensity and (**B**) CXCR4-positive cell population. Silencing was assessed after 2 and 3 days of CXCR4-specific siRNA or control siRNA treatment (50 nM with polymer:siRNA ratio of 4∶1). The polymers used were PEI25, PEI2-LA (2.1 LA/PEI) and PEI2-CA (6.9 CA/PEI).

Time-dependent siRNA delivery was then assessed by utilising only the polymers with the highest substitution levels at the best performing ratio (8∶1) and exposing the complexes to cells for a period of up to 48 hours (**Figure 9**). The highest siRNA delivery was obtained at the earliest time point (6 h). As time increased, the percentage of cells associated with complexes and the mean fluorescence/cell was decreased. The mean fluorescence levels declined at a faster rate than the percentage of siRNA-positive cells. The decline was most likely a result of rapid cell proliferation, as the doubling-rate of THP-1 cells is ∼26 h and KG-1 and HL-60 cells being slightly longer. In such a case, a rapid drop in the levels of siRNA concentrations is expected, whereas the percentage of siRNA-positive cells might not change drastically. The latter will be seen for cells where the siRNA amounts were lower (i.e., at the detection threshold). As the variation of mean fluorescence (given by standard deviations in **Figure 9**) did not increase with time, it is reasonable to assume that the splitting of complexes between the dividing cells were fairly even with internalized siRNA not particularly restricted to mother or daughter cells. Even with the prolonged time of analysis, both PEI25 and PEI2 did not yield a significant siRNA delivery, based on the mean siRNA fluorescence associated with the cell population (**Figure 9ii**). Collectively, these results indicated that the uptake of the siRNA complexes was relatively rapid (<6 h) and prolonged incubation with complexes did not yield a ‘net’ accumulation of siRNA inside cells.

As the interaction with serum proteins can affect delivery of siRNA complexes, we explored the effect of serum on siRNA delivery, utilizing the best performing polymer PEI2-LA (**Figure 10**). At low serum percentage of 2.5%, PEI25 displayed comparable delivery percentage to PEI2-LA, although the siRNA delivery/cell remained low as usual for this polymer. However, as the serum concentration was increased, the delivery ability of PEI25 decreased to a greater degree than the PEI2-LA in all cell lines. In THP-1 cells, the effect of serum on PEI2-LA delivery was evident even at low concentration (i.e., from 2.5 to 10%), whereas delivery was largely unaffected up to 25% serum in the KG-1 and HL-60 cells. Although, the polymer-serum interactions were expected to produce similar results in all cell lines as the serum percentage and polymer remain the same, properties that promote of uptake, can be dependent on the cell type. The ability to deliver siRNA in serum is clearly important especially for leukemia cells, however similar uptake studies in the presence of serum have not been reported in literature for leukemic cells. Clearly PEI25 was affected by the serum proteins to a much greater extent than the lipid-modified PEI2. We previously found that lipid substituted PEIs afforded better protection against degradation in the presence of serum [40]. This observation along with the uptake results suggest that the lipid modification decreased interaction of complexed nucleic acids with serum proteins, so that their delivery ability is less affected by high serum concentrations.

### 3.6 Comparison of siRNA Delivery with Commercial Reagents

The best performing polymeric carrier, PEI2-LA, was next compared to several commercial reagents for siRNA delivery to AML cells. The commercial reagents were chosen based on their previous use for siRNA delivery to leukemic cells (see Introduction). All of the commercial reagents were cationic liposomes, since no polymeric carrier was utilized in leukemic cells previously. The siRNA delivery was investigated at the polymer:siRNA ratios of 8∶1 and 4∶1, but the results from the 8∶1 ratio is shown only since the results from the 4∶1 ratio also gave equivalent outcomes. The results were summarized as either the percentage of siRNA-positive cells (**Figure 11A**), or the extent of siRNA delivery per cell (**Figure 11B**). The siRNA delivery by the chosen reagents varied significantly, depending on the cell line. Fugene HD, PEI25, Metafectamine and PEI2-LA were the carriers with the highest delivery percentages (>80%; **Figure 11A**), whereas Lipofectamine™ 2000, RNAiMAX, DOTAP and HiPerFect displayed variable results depending on the cell line. Based on the mean fluorescence levels, Metafectamine, Lipofectamine™ 2000 and PEI2-LA were the top three carriers (**Figure 11B**), but RNAi-mate and DOTAP did not demonstrate significant delivery at all. The polymeric PEI2-LA was among the top three carriers when ranked in both the mean siRNA fluorescence (**Figure 11C**, **top**) and the percentage of siRNA-positive cell population (**Figure 11C**, **bottom**). The ranking was not consistent with the two parameters assessed, indicating that the individual carriers behaved differently in the extent of modification and the mean siRNA delivered into each cell type. We noted a significant variation in siRNA delivery among the three cell types for these different carriers, as noted in **Figure 9** as well.

There has not been any comparison of the efficiency of commercial reagents for siRNA delivery to leukemic cells, but it is clear that large differences in efficiency existed among these reagents. Lipofectamine™ 2000, Metafectamine and Fugene HD seemed to be sufficiently effective when one wishes to employ an ‘off-the-shelf’ siRNA delivery system. In performing this analysis, we attempted to follow the manufacturer’s recommendations for optimal reagent:siRNA ratio, and employed a single complexation buffer for all reagents. It is likely that the efficiency for some reagents may be improved with further optimization of complexation conditions, however such an effort was not spent in this study due to extensive numbers of variables that can be optimized. Our main goal was to identify a few obviously effective commercial reagents and to compare our polymeric carriers to these reagents in silencing studies (below).

### 3.7 Silencing of Reporter (GFP) Gene Expression

To explore the silencing efficiency of the developed polymers, GFP-expressing THP-1 cells were used as a model system. The reduction in GFP expression was expressed as either a percent decrease in mean GFP fluorescence or percent decrease in GFP-positive cells (**Figure 12**). Note that the extent of GFP expression in the modified THP-1 cells was typically 3 logs higher than the background in unmodified cells (unmodified cells appeared in first quadrant of the histograms; not shown). The GFP silencing was evident by a leftward shift in the histograms (see **Figure 12A**), but at no point complete GFP silencing was obtained in this study. The control siRNA employed led to minor (insignificant) changes in GFP fluorescence of the cells at times, so that the changes in GFP fluorescence was normalized against CsiRNA treated cells (as described in Methods). The initial study focussed on comparing the PEI25 to two of the lipid substituted polymers (PEI2-CA and PEI2-LA) with higher siRNA delivery efficiency to THP-1 cells (from **Figure 9**). At the three polymer:siRNA ratios tested (2∶1, 4∶1 and 8∶1), both lipid substituted polymers gave a higher silencing activity than PEI25, whose silencing efficacy was not significant (**Figure 12B** **)**. Unlike the delivery results, where LA substitution gave the most delivery, CA substitution was generally more effective in GFP silencing, with 20–26% GFP silencing achieved after 3 days of siRNA delivery. A dose-response curve was then explored with the lipid-substituted polymers at 4∶1 and 8∶1 polymer:siRNA ratios (**Figure 12C**). When silencing was based on mean GFP fluorescence, a gradual increase in GFP silencing was evident for some groups between 20 and 100 nM siRNA concentration (e.g., for CA and LA substituted PEI2 at 8∶1 ratio), but not beyond the 100 nM siRNA concentration. Such clear dose-response curves were not evident for silencing based on percentage of GFP-positive cells (**Figure 12C**).

The silencing efficiency of the polymeric carriers (PEI2-LA and PEI2-CA) and three effective commercial reagents (Lipofectamine™ 2000, Metafectamine and Fugene HD) were compared next (**Figure 12D**). GFP silencing was assessed over a period of 9 days after a single siRNA delivery. Although PEI25 was also included in this experiment, only a small fraction of cells (<5%) survived on the long run so that it was omitted from the analysis. The siRNA delivery resulted in most significant GFP silencing on day 3 for all delivery systems, and silencing was typically lost by 9 days (**Figure 12D**). Lipofectamine™ 2000 gave the highest silencing (∼62% on day 3 based on mean GFP fluorescence), followed by the two lipid-substituted PEIs and Metafectamine (∼40% on day 3). Similar conclusions were reached when GFP silencing was analysed based on the decrease in GFP-positive cells.

However, a major difference was seen in cell numbers analysed by the flow cytometry; (i) while LA substituted PEI2 did not give any long term adverse effects on cells (i.e., cell numbers were equivalent to no-treatment controls), CA substituted PEI2 gave lower cell numbers especially after day 3; (ii) Lipofectamine™ 2000 in particular resulted in gradual loss of cell survival to levels <5% of the un-treated cells, indicating long terms adverse effects on the cells, and (iii) Metafectamine and Fugene HD gave intermediate effects on the cells, where the cell numbers typically remained at the ∼50% level to that of no-treatment controls. The long-term adverse effects on cells are obviously not desirable for systemic administration of delivery systems due to undesirable effects on healthy cells. The difference in the toxicity of CA and LA substituted PEI2 was noteworthy in the silencing studies and was not apparent in the initial studies (see **Figure 4**). The silencing studies employed cell concentrations from flow cytometry as a measure of toxicity, whereas the initial toxicity studies investigated cell viability by the MTT. While the results from CA substitutions agreed with both methods, results with LA substitution did not agree between the two methods. This issue requires further investigation but it appears that LA substitution seems to be more desirable for longer exposure to the cells. Despite effective silencing, the toxicity of Lipofectamine™ 2000 was considered prohibitive for *in vitro* use (since aberrant cellular physiology could complicate the investigated silencing phenomena) as well as *in vivo* use (too toxic for non-target cells and tissues). Such high toxicities were not evident in previous studies employing this reagent [11]–[16], since these studies were more concerned with elucidating the biological roles of specific targets, rather than safety and efficacy of the delivery system.

To confirm whether the silencing observed with GFP-positive cells also reflected silencing at the mRNA level, GFP mRNA levels in treated THP-1 cells was quantitated by PCR. A significant decrease in mRNA levels was observed with PEI2-CA delivered siRNA on day 1 and 2, after which insignificant change was seen on day 3 (**Figure 13A**). The cells exposed to PEI25 and PEI2-LA delivered siRNA did not yield as significant silencing at the mRNA level (**Figure 13A**). As before, silencing was additionally confirmed based on changes in GFP-positive cells and mean GFP levels, especially with PEI2-CA (**Figure 13B** **i and 13Bii**, respectively). It appeared that flow-cytometric assessment of GFP silencing was more readily detectable as compared to PCR-based assessment, given large variations observed with the latter assay. However, both PCR and flow cytometric based evaluation of silencing suggested the CA-substituted polymers to be more effective in functional siRNA delivery.

### 3.8. Down-regulation of Endogenous CXCR4 Levels

The G protein-coupled chemokine receptor CXCR4 is an endogenous protein that has implications in abnormal proliferation of leukemic cells, migration and anchorage to the bone marrow [56] and with differential expression as a response to drug treatments including valproic acid (VPA) depending on maturation level of the cells [57]. Since THP-1 cells display high level of CXCR4 expression (>80% positive), we explored the feasibility of down-regulating the level of this endogenous protein since silencing CXCR4 may prove beneficial in leukemia treatments. After treatment with 50 nM CXCR4-specific siRNA in THP-1 cells, a significant decrease in the mean CXCR4 level was achieved at day 2 with PEI2-CA and by day 3 with PEI2-LA (**Figure 14A**). Similar to the GFP silencing results, the most effective polymer was PEI2-CA, however, PEI2-LA was only slightly less efficient and PEI25 was not effective in this case. The extent of maximal decrease in the CXCR4 levels was 20–30%, a value similar to the extent of silencing observed with the GFP. A decrease in CXCR4-positive cell population occurred with a decrease of 8.9% for PEI2-LA20 and 6.8% for PEI2-CA20 (**Figure 14B**). Unlike the GFP, CXCR4 is highly dynamically regulated [57], [58] and it is possible that rapid regulation of CXCR4 levels could effect silencing in the cell population.

Considering all silencing results, it appeared that the designed polymers gave effective silencing (up to ∼35% based on GFP silencing and ∼30% based on CXCR4 levels) between 25 and 50 nM siRNA concentration and the benefit of employing higher siRNA concentrations was not immediately evident. Non-specific effects of siRNA treatment were investigated previously [59], [60]. Persengiev et al. reported an increase as well as a decrease in the expression of various mammalian genes in response to a luciferase siRNA treatment (where no natural target is expected to exist). They observed a concentration-dependent effect of siRNA in various genes with siRNA concentrations at >25 nM [59]. Semizarov et al. also observed off-target effects of siRNA at 100 nM, but not at 20 nM [60]. Therefore, the delivery formulations developed in this study appear to function favourably considering this constraint. Being a reporter protein, GFP silencing was not expected to lead to any functional changes and silencing specific targets for desirable functional changes are under study at the present time. CXCR4-silencing, on the other hand, are expected to yield several functional outcomes, such as reduced migration, abnormal proliferation and reduction in cellular anchorage to bone marrow [57], and thereby reduced cell survival. These effects will be the focus of further studies. We noted that previous silencing studies with leukemic cells rarely reported quantitative results due to the mechanistic nature of the studies. Some studies reported quantitative silencing outcomes; (i) >80% and >95% FADD protein silencing with Lipofectamine™ 2000 (100 nM siRNA) in U937 [14] and K562 [15] cells, (ii) ∼90% E2F1 protein silencing in Jurkat T-cells with HiPerFect (siRNA concentration not specified) [19], ∼70% BCR-ABL protein silencing with Lipofectamine™ 2000 (60–180 nM siRNA) and a Tat–LK15 peptide in K562 cells [16], and (iv) ∼60% *Bcr-abl* mRNA with DOTAP (54 pM siRNA – an exceptionally low dose) in CML cells [20]. Our silencing results were not as high as these values, but it must be pointed out that our cells had exceptionally high GFP levels and complete silencing was not considered a realistic goal with this protein. The CXCR4 down-regulation, however, was not also as high as the values reported by other groups. The results from Lipofectamine™ 2000 mediated silencing in this study as compared to other studies might serve as a good indicator of the differences in cell models used for silencing. The silencing efficiency in this study should be considered for comparison purposes for efficacy and toxicity among the carriers, and not in the context of therapeutic studies. Such studies with relevant molecular targets (including CXCR4) are currently underway in the authors’ labs.

Finally, we explored the utility of our polymers for silencing in two additional cell types, the Hut78 cells (a T-cell lymphoma cell line) and the K562 cells (a chronic myeloid leukemia cell line) using the reporter GFP as the target. These cells display constitutive GFP expression similar to the THP-1 cells extensively used in this study. Preliminary results indicated the feasibility of GFP silencing in the Hut78 cells, most effectively with PEI2-LA (**Figure S3A**). The silencing in K562 cells was to a lesser extent with PEI2-LA, suggesting that the performance of the developed polymeric systems could depend on the specific cell type. Such a differential performance was also evident with the PEI25 mediated GFP-specific siRNA (**Figure S3B**), where performance of PEI25 in K562 cells was superior to the Hut78 cells.

### Conclusions

Lipid modification of the ineffective polymer PEI2 clearly improved its ability to deliver siRNA to leukemic cells. Enhanced siRNA delivery was obtained with the appropriate choice of lipid for polymer substitution, whose delivery ability was dependent on (i) lipid substitution levels and (ii) polymer:siRNA ratio used for complex formation. Compared to the commonly used polymeric carrier PEI25, the leading lipid-substituted polymer PEI2-LA gave siRNA delivery that was less dependent on serum concentration in medium. Among the three types of AML cells explored, cell-to-cell differences in siRNA delivery was evident, suggesting that optimization of siRNA complex formulations might be needed to maximize the delivery for individual cell types. The PEI2-LA compared favourably to several commercial siRNA delivery systems, and provided an effective silencing activity without significantly affecting the subsequent cell growth. Silencing was demonstrated by using a reporter (GFP) gene as well as the endogenous protein CXCR4 in THP-1 cells. Given the fully disclosed nature of the PEI2-LA (unlike most commercial reagents) and the versatility of polymeric delivery systems in general, the proposed polymers provide excellent possibilities for therapeutic delivery of siRNA in leukemia. The polymers could also serve as a platform to further improve siRNA delivery by incorporating functional groups such as cell targeting ligands and moieties facilitating endosomal release. The in vivo efficacy of the polymeric siRNA delivery systems was not explored in this study and it will be the next stage in evaluation of the proposed delivery systems.

## Supporting Information

Figure S1 Effect of complexation time on siRNA delivery. PEI2-LA20 was delivered after complexes were prepared and incubated at room temperature from 0–120 minutes.
(PDF)

Figure S2 Effect of free fatty acids on siRNA-polymer delivery. (**A**) Fatty acids were pre-treated for 24 h with LA and then incubated with FAM siRNA/polymer complexes (1:2 and 1∶8 polymer:siRNA ratios) for 24 h. (**B**) Various fatty acids were delivered with siRNA-polymer (25 nM at 1∶8 polymer:siRNA ratio) treatments simultaneously to THP-1 cells for 24 h.
(PDF)

Figure S3 GFP Silencing in GFP-Positive Hut78 (**A**) and K562 (**B**) cells. GFP silencing was measured 3 days after siRNA treatment with 25 nM (Hut78 cells) and 36 nM (K562) GFP siRNA (or control siRNA) at indicated polymer:siRNA ratios. Percent decrease in GFP-positive cells are indicated in the top graphs whereas percent decrease in the mean GFP levels are indicated in bottom graphs.
(PDF)

Table S1 Lipid substituted 2 kDa PEI library (PEI2-Lipids).
(DOCX)

Table S2 Linear regression analysis of complex cytotoxicity.
(DOCX)

Table S3 Trends between complex cytotoxicity and lipid substitutions.
(DOCX)
