# Supplementary figures and images for: Effective Non-Viral Delivery of siRNA to Acute Myeloid Leukemia Cells with Lipid-Substituted Polyethylenimines
Source: PLoS One. 2012 Aug 31;7(8):e44197. doi: 10.1371/journal.pone.0044197 (PMC3432090; doi:10.1371/journal.pone.0044197)

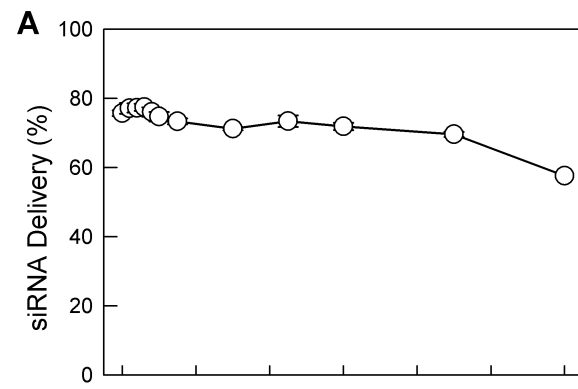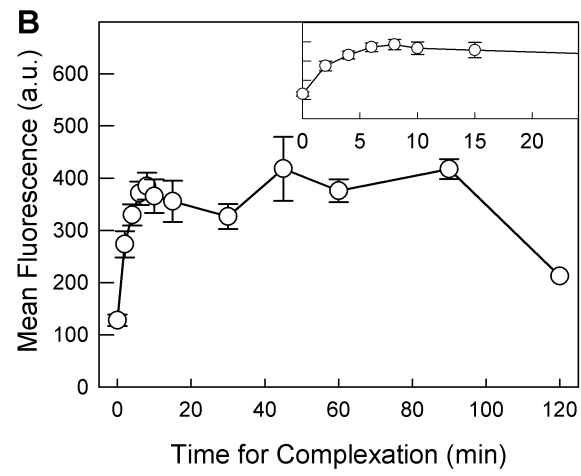

Supplement: Figure S1 — Effect of complexation time on siRNA delivery. PEI2-LA20 was delivered after complexes were prepared and incubated at room temperature from 0–120 minutes. (PDF) [file pone.0044197.s001.pdf]

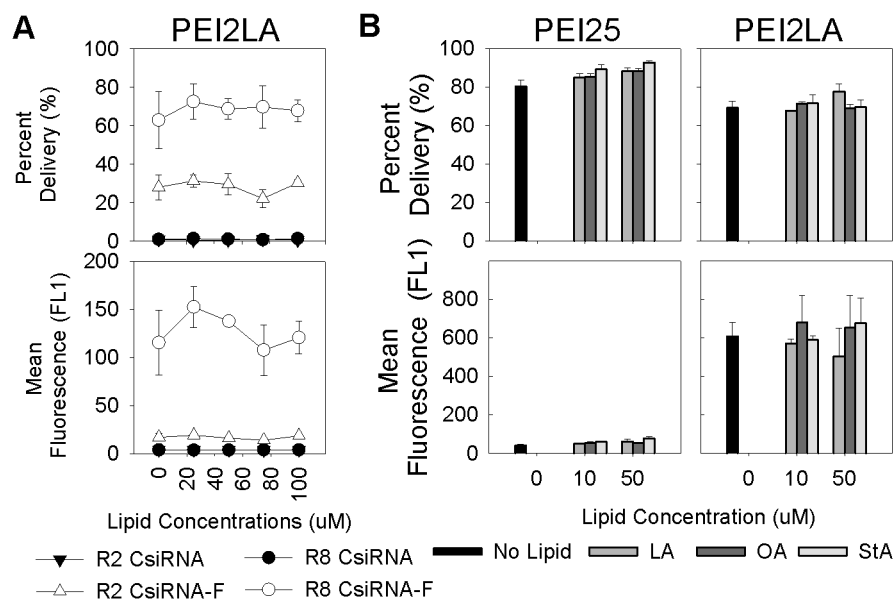

Supplement: Figure S2 — Effect of free fatty acids on siRNA-polymer delivery. (A) Fatty acids were pre-treated for 24 h with LA and then incubated with FAM siRNA/polymer complexes (1:2 and 1∶8 polymer:siRNA ratios) for 24 h. (B) Various fatty acids were delivered with siRNA-polymer (25 nM at 1∶8 polymer:siRNA ratio) treatments simultaneously to THP-1 cells for 24 h. (PDF) [file pone.0044197.s002.pdf]

### A GFP+ HUT78

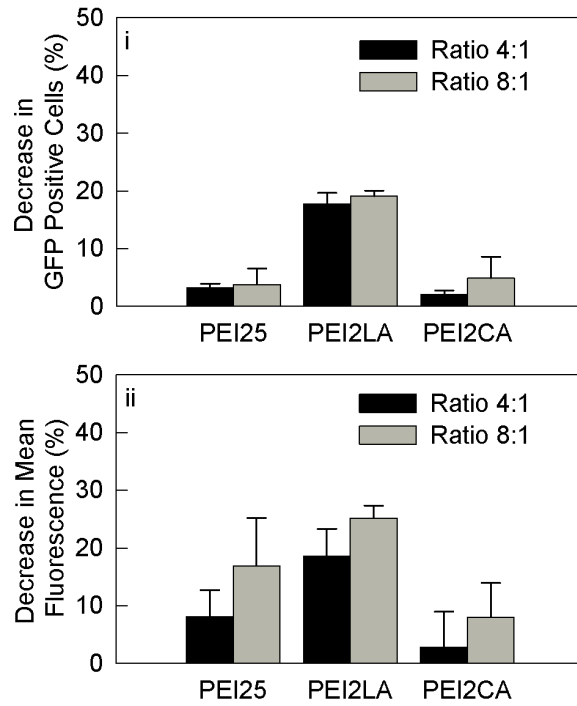

### B GFP+ K562

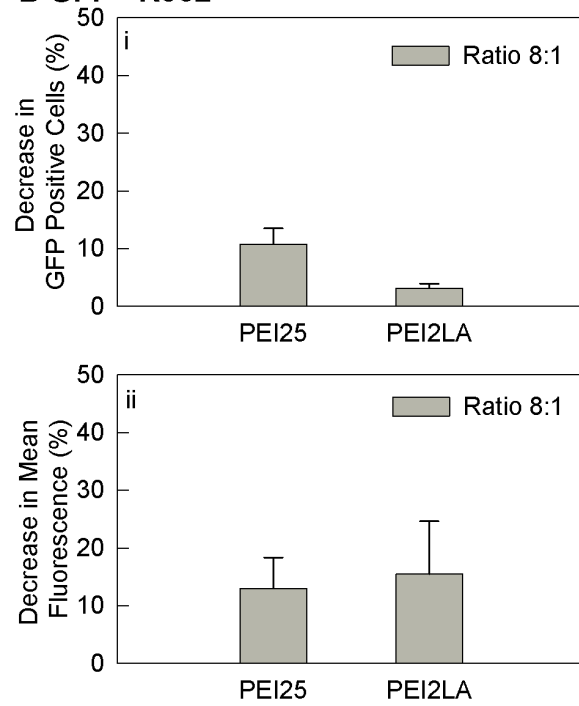

Supplement: Figure S3 — GFP Silencing in GFP-Positive Hut78 (A) and K562 (B) cells. GFP silencing was measured 3 days after siRNA treatment with 25 nM (Hut78 cells) and 36 nM (K562) GFP siRNA (or control siRNA) at indicated polymer:siRNA ratios. Percent decrease in GFP-positive cells are indicated in the top graphs whereas percent decrease in the mean GFP levels are indicated in bottom graphs. (PDF) [file pone.0044197.s003.pdf]
