# Supplementary material for: Effective Non-Viral Delivery of siRNA to Acute Myeloid Leukemia Cells with Lipid-Substituted Polyethylenimines
Source: PLoS One. 2012 Aug 31;7(8):e44197. doi: 10.1371/journal.pone.0044197 (PMC3432090; doi:10.1371/journal.pone.0044197)
Supplement: Table S1 — Lipid substituted 2 kDa PEI library (PEI2-Lipids). (DOCX) [file pone.0044197.s004.docx]

| Polymer | Substitution | Feed Ratio | Lipid/PEI^a^ | Methylene/  PEI^b^ | % Substitution^c^ |
| --- | --- | --- | --- | --- | --- |
| PEI2-CA0.1 | Caprylic Acid (CA)  C8:0 | 0.012 | 0.5 | 3.7 | 2.9 |
| PEI2-CA1 |  | 0.066 | 1.1 | 8.8 | 6.9 |
| PEI2-CA10 |  | 0.100 | 2.4 | 19.0 | 14.8 |
| PEI2-CA20 |  | 0.200 | 6.9 | 56.8 | 43.4 |
| PEI2-PA0.1 | Palmitic Acid (PA)  C16:0 | 0.012 | 0.3 | 4.9 | 1.9 |
| PEI2-PA1 |  | 0.066 | 0.6 | 9.5 | 3.7 |
| PEI2-PA10 |  | 0.100 | 0.8 | 12.6 | 4.9 |
| PEI2-PA20 |  | 0.200 | 1.1 | 18.0 | 7.0 |
| PEI2-OA0.1 | Oleic Acid (OA)  C18:1 | 0.012 | 0.3 | 4.6 | 1.6 |
| PEI2-OA1 |  | 0.066 | 1.0 | 18.1 | 6.3 |
| PEI2-OA10 |  | 0.100 | 1.7 | 30.0 | 10.4 |
| PEI2-OA20 |  | 0.200 | 2.5 | 44.1 | 15.3 |
| PEI2-LA0.1 | Linoleic Acid (LA)  C18:2 | 0.012 | 0.2 | 4.3 | 1.5 |
| PEI2-LA1 |  | 0.066 | 1.0 | 17.3 | 6.0 |
| PEI2-LA10 |  | 0.100 | 1.8 | 33.2 | 11.5 |
| PEI2-LA20 |  | 0.200 | 3.2 | 57.7 | 20.0 |
| PEI2-StA0.1 | Stearic Acid (StA)  C18:0 | 0.012 | 0.2 | 3.2 | 1.1 |
| PEI2-StA1 |  | 0.066 | 0.5 | 8.4 | 2.9 |
| PEI2-StA10 |  | 0.100 | 3.6 | 66.6 | 22.8 |
| PEI2-StA20 |  | 0.200 | 4.9 | 89.0 | 30.9 |
| PEI2-MA0.1 | Myristic Acid (MA)  C14:0 | 0.012 | 0.4 | 5.2 | 2.3 |
| PEI2-MA1 |  | 0.066 | 0.6 | 8.3 | 3.7 |
| PEI2-MA10 |  | 0.100 | 1.7 | 24.1 | 10.8 |
| PEI2-MA20 |  | 0.200 | 1.5 | 20.8 | 9.3 |

a. Actual number of lipids substituted per PEI2 (calculated from ^1^H-NMR analysis).

b. Lipid carbon (C) substitutions per PEI2 were calculated based on the number of Cs present in each lipid and the number of lipids substituted per PEI2.

c. Percent substitution refers to the percentage of primary amines modified with the corresponding lipids.
