# Supplementary material for: Effective Non-Viral Delivery of siRNA to Acute Myeloid Leukemia Cells with Lipid-Substituted Polyethylenimines
Source: PLoS One. 2012 Aug 31;7(8):e44197. doi: 10.1371/journal.pone.0044197 (PMC3432090; doi:10.1371/journal.pone.0044197)
Supplement: Table S2 — Linear regression analysis of complex cytotoxicity. (DOCX) [file pone.0044197.s005.docx]

| Polymer | THP-1 |  | KG-1 | | HL60 | |
| --- | --- | --- | --- | --- | --- | --- |
|  | r^2 a^ | P^b^ | r^2a^ | P^b^ | r^2a^ | P^b^ |
| PEI25 | **0.9537** | **0.0234** | **0.9641** | **0.0181** | **0.9845** | **0.0078** |
| PEI2 | 0.1693 | 0.5885 | **0.6821** | 0.1737 | 0.8850 | 0.0593 |
| CA1 | **0.9987** | **0.0007** | 0.0005 | 0.9778 | 0.5822 | 0.2370 |
| CA10 | **0.8166** | 0.0964 | 0.2286 | 0.5219 | **0.9866** | **0.0067** |
| CA20 | **0.9930** | **0.0035** | **0.9200** | **0.0408** | **0.9701** | **0.0151** |
| PA1 | **0.8948** | 0.0541 | 0.07418 | 0.7276 | **0.9550** | **0.0228** |
| PA10 | 0.7470 | 0.1357 | 0.04244 | 0.7940 | 0.1294 | 0.6403 |
| PA20 | **0.9571** | **0.0217** | 0.1482 | 0.6151 | **0.9898** | **0.0051** |
| OA1 | 0.3485 | 0.4096 | 0.4284 | 0.3455 | **0.9924** | **0.0038** |
| OA10 | 0.2134 | 0.5380 | 0.03309 | 0.8181 | **0.9252** | **0.0381** |
| OA20 | 0.4570 | 0.3240 | 0.2687 | 0.4816 | 0.6200 | 0.2125 |
| LA1 | **0.9133** | **0.0444** | **0.9964** | **0.0018** | 0.8184 | 0.0954 |
| LA10 | **0.9193** | **0.0412** | **0.8908** | 0.0562 | **0.9327** | **0.0343** |
| LA20 | 0.8408 | 0.0831 | **0.7874** | 0.1126 | **0.9951** | **0.0024** |

a. Linear regression r^2^ values

b. Calculated to determine if slope was significantly different from zero

* Significant values are bolded.
