# Supplementary material for: Effective Non-Viral Delivery of siRNA to Acute Myeloid Leukemia Cells with Lipid-Substituted Polyethylenimines
Source: PLoS One. 2012 Aug 31;7(8):e44197. doi: 10.1371/journal.pone.0044197 (PMC3432090; doi:10.1371/journal.pone.0044197)
Supplement: Table S3 — Trends between complex cytotoxicity and lipid substitutions. (DOCX) [file pone.0044197.s006.docx]

| Concentration  (µg/ml) | Lipid  Substituted | THP-1 | | KG-1 | | HL60 | |
| --- | --- | --- | --- | --- | --- | --- | --- |
|  |  | r^2 a^ | P^b^ | r^2a^ | P^b^ | r^2a^ | P^b^ |
| 10 | ALL | 0.05520 | 0.4397 | 0.2776 | 0.0643 | 0.1185 | 0.2493 |
|  | CA | **0.9790** | **0.0106** | **0.9621** | **0.0191** | **0.5470** | 0.2604 |
|  | PA | **0.9123** | **0.0449** | **0.7009** | 0.1628 | **0.8228** | 0.0929 |
|  | OA | 0.4665 | 0.3170 | 0.1190 | 0.6551 | 0.0056 | 0.9249 |
|  | LA | **0.6978** | 0.1647 | 0.0448 | 0.7884 | 0.1150 | 0.6609 |
| 5 | ALL | 0.1119 | 0.7309 | 0.1837 | 0.1440 | 0.0006 | 0.9364 |
|  | CA | **0.8374** | 0.0849 | **0.9179** | **0.0419** | 0.1876 | 0.5669 |
|  | PA | **0.6975** | 0.1648 | 0.4946 | 0.2967 | **0.5650** | 0.2483 |
|  | OA | 0.4842 | 0.3042 | **0.9321** | **0.0346** | 0.0454 | 0.7869 |
|  | LA | **0.6464** | 0.1960 | 0.1360 | 0.6312 | 0.0575 | 0.7602 |

a. Linear regression r^2^ values

b. Calculated to determine if slope was significantly different from zero

* Significant values are bolded.
